# Substrate specificity of 2-deoxy-D-ribose 5-phosphate aldolase (DERA) assessed by different protein engineering and machine learning methods

**DOI:** 10.1007/s00253-020-10960-x

**Published:** 2020-11-04

**Authors:** Sanni Voutilainen, Markus Heinonen, Martina Andberg, Emmi Jokinen, Hannu Maaheimo, Johan Pääkkönen, Nina Hakulinen, Juha Rouvinen, Harri Lähdesmäki, Samuel Kaski, Juho Rousu, Merja Penttilä, Anu Koivula

**Affiliations:** 1 VTT Technical Research Centre of Finland Ltd, P.O. Box 1000, FI-02044 VTT, Espoo, Finland; 2 Department of Computer Science, Aalto University, Espoo, Finland; 3 Helsinki Institute for Information Technology, Espoo, Finland; 4 Department of Chemistry, University of Eastern Finland, PO Box 111, FI-80101 Joensuu, Finland

**Keywords:** DERA, Aldolase, Protein engineering, Machine learning, Crystal structure determination, C–C bond formation, Biocatalysis

## Abstract

**Abstract:**

In this work, deoxyribose-5-phosphate aldolase (*Ec* DERA, EC 4.1.2.4) from *Escherichia coli* was chosen as the protein engineering target for improving the substrate preference towards smaller, non-phosphorylated aldehyde donor substrates, in particular towards acetaldehyde. The initial broad set of mutations was directed to 24 amino acid positions in the active site or in the close vicinity, based on the 3D complex structure of the *E. coli* DERA wild-type aldolase. The specific activity of the DERA variants containing one to three amino acid mutations was characterised using three different substrates. A novel machine learning (ML) model utilising Gaussian processes and feature learning was applied for the 3rd mutagenesis round to predict new beneficial mutant combinations. This led to the most clear-cut (two- to threefold) improvement in acetaldehyde (C2) addition capability with the concomitant abolishment of the activity towards the natural donor molecule glyceraldehyde-3-phosphate (C3P) as well as the non-phosphorylated equivalent (C3). The *Ec* DERA variants were also tested on aldol reaction utilising formaldehyde (C1) as the donor. *Ec* DERA wild-type was shown to be able to carry out this reaction, and furthermore, some of the improved variants on acetaldehyde addition reaction turned out to have also improved activity on formaldehyde.

**Key points:**

• *DERA aldolases are promiscuous enzymes.*

• *Synthetic utility of DERA aldolase was improved by protein engineering approaches.*

• *Machine learning methods aid the protein engineering of DERA.*

**Supplementary Information:**

The online version of this article (10.1007/s00253-020-10960-x) contains supplementary material, which is available to authorized users.

## Introduction

**Scheme 1:**
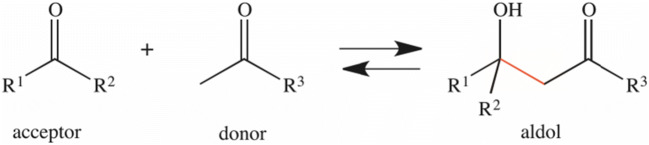
Aldolases catalyse the reversible formation of C–C bonds by the aldol addition of a nucleophilic donor, typically a ketone enolate, onto anelectrophilic aldehyde acceptor

Aldolases are enablers of industrial biocatalysis as they can promote carbon-carbon (C–C) bond formation, which is one of the essential reactions in synthetic chemistry. Aldol reaction can be catalysed by the lyase (EC4) or transferase class (EC2) of enzymes, found in the metabolic pathways in all three domains of life (archaea, bacteria, eukarya). Aldolases catalyse the reversible formation of C–C bonds by the aldol addition of a nucleophilic donor, typically a ketone enolate, onto an electrophilic aldehyde acceptor (Scheme 1).

Aldolase type of enzymes have been found to be promiscuous capable of using a broad range of aldehydes as acceptors, whereas the donor compound is often structurally invariable. Hence, aldolases can be classified according to their donor specificity to, e.g. acetaldehyde-dependent aldolases. Another way to classify these enzymes relates to the different catalytic mechanisms to activate the nucleophilic component. The class I aldolases do not require any cofactor, but exhibit a conserved lysine residue in the active site which forms a Schiff base intermediate with the donor compound to generate an enamine nucleophile.

Deoxyribose-5-phosphate aldolases (DERA, EC 4.1.2.4) are a class I aldolase that catalyses in vivo the reversible addition of the donor molecule, acetaldehyde (C2), to the acceptor molecule, glyceraldehyde 3-phosphate (C3P). DERA is known to be promiscuous in its substrate range as it accepts a wide variety of different acceptor molecules (Chen et al. 1992; Gijsen and Wong 1994). In addition, at the donor site of DERA enzyme acetone, fluoroacetone and propionaldehyde (propanal) have been reported to function (with strongly reduced reaction rates) besides the acetaldehyde (Barbas et al. 1990; Chen et al. 1992). An interesting and unique feature, particularly in terms of synthesis reactions, is the ability of DERA to catalyse sequential acetaldehyde addition (Gijsen and Wong 1994). Here, the first aldol addition reaction creates an aldehyde product, which functions as an acceptor for the subsequent DERA-catalysed stereoselective aldol reaction to add another aldehyde donor substrate. This cascade reaction has been utilised for addition of two equivalents of acetaldehyde to one equivalent of chloroacetaldehyde in the preparation of (3R,5S)-6-chloro-2,4,6-trideoxyhexose, a chiral precursor for the sidechain of the statin drugs (Oslaj et al. 2013). The capacity of wild-type DERA to catalyse aldol addition is, however, rather low. To increase the affinity of the *E. coli* aldolase for chloroacetaldehyde acceptor and stability against high acetaldehyde concentrations, researchers at DSM applied a directed evolution approach (Jennewein et al. 2006). By screening for and combining beneficial mutations, they succeeded in identifying an improved variant with 10-fold improved productivity in *E. coli* under industrially relevant conditions.

Advances in computational chemistry combined with protein engineering strategies have most recently opened new possibilities for more efficient design of enzymatic catalysts for chemical reactions (Linder 2012; Kiss et al. 2013; Mak and Siegel 2014; Jindal et al. 2019). The aim of this work was to engineer DERA aldolase to accept smaller non-phosphorylated acceptor substrates in the aldol addition reaction with acetaldehyde. The work included evaluating first the most suitable DERA aldolase for the protein engineering work, by expressing, purifying and characterising a set of DERA enzymes of different origin. After that, we used different types of mutagenesis approaches in combination with a novel machine learning model to create DERA variants in three mutagenesis rounds, which were screened using a panel of different substrates. Crystal structures of some of the most interesting DERA variants were also solved to provide more insight.

## Materials and methods

### Cloning, protein expression and purification of different DERAs

Seven DERA encoding genes from different organisms (1. DERA [UniProt P0A6L0]-coding gene [deoC] from *E. coli*, 2. DERA [NCBI Reference Sequence: WP_047758083.1] -coding gene from *Geobacillus*, 3. DERA [SwissProt Q5SJ28] -coding gene from *Thermus thermophilus*, 4. DERA [UniProt E0CX06] -coding gene from *Coccidioides immitis*, 5. DERA [UniProt Q03Q50]-coding gene from *Lactobacillus brevis*, 6. DERA [GenBank CRG82919.1]-coding gene from *Talaromyces islandicus* and 7. DERA [NCBI Reference Sequence: XP_003188826.1]-coding gene from *Aspergillus niger*) were cloned and expressed in *E. coli.* All of them were codon optimised for *E. coli* and synthesised with an N-terminal 6x His-tag by Integrated DNA Technologies as so called G-blocks. Histidine tag was added to the N-terminus as it has been shown that the C-terminal tail of *Ec*DERA has a role in the catalytic activity (Schulte et al. 2018). For the codon-optimised nucleotide sequences, see “GenBank accession numbers” section. The synthetic DNA blocks were cloned into the pBAT4 vector (Peränen et al. 1996) linearised with *Nco*I and *Xho*I restriction enzymes. The synthesised insert contained 60 bp overlapping regions in both 5′ and 3′ ends to the vector to allow cloning with Gibson assembly method (Gibson et al. 2009) using Gibson assembly® master mix (New England Biolabs). After assembly, the mixtures were transformed into chemical competent XL1-blue *E. coli* cells.

Single point mutations of *E. coli* DERA (*Ec* DERA) were made with Q5®site-directed mutagenesis kit (New England Biolabs) (list of primers is shown in Table S1) and verified by sequencing (Source BioScience or Microsynth). Some amino acid positions were mutated by using degenerated primers to generate a selection of amino acid mutations in one PCR reaction. For example, position L20 was mutagenised using forward primer 5′-GTTGATGGACSDNACCACTCTGAACG-3′ to generate mutations L20R, Q, E, H, V, D, G simultaneously. Nucleotide code S stands for G or C and D stands for A, G or T.

Double mutants (containing two point mutations/gene) and some of the triple mutants (containing three point mutations/gene) of *Ec* DERA were generated in a similar manner as for the single mutants by using already existing single mutant in question as the template in the PCR mutagenesis. The triple mutant variants suggested by machine learning were ordered as synthetic DNA blocks from Integrated DNA Technologies, similarly to the wild-type DERA genes described above. Saturation mutagenesis was done by PCR using so called 22c-trick (Kille et al. 2013). The method reduces codon redundancy from often-used saturation mutagenesis method using codon NNK.

DERA variants were expressed in *E. coli* BL21(DE3) strain in LB-medium containing 100 μg/ml ampicillin. DERA wild-type enzymes from different organisms and *Ec* DERA mutants N21K and triple mutant C47V/G204A/S239D, which were crystallised for 3D structure determination, were cultivated in 50-ml scale and all other *Ec* DERA mutants were cultivated in 3-ml or 10-ml scale. The expression strains were cultivated for 6–8 h in 37 °C after which the expression was induced with 0.5 mM IPTG, and after 16-h incubation at 30 °C, cells were harvested (10 min at 4000×*g*). For cell lysis, the cells were re-suspended in B-PER Bacterial Protein Extraction Reagent (Thermo Scientific) supplemented with protease inhibitor (cOmplete mini, EDTA-free, Roche), lysozyme (Sigma Aldrich), and DNAse (Roche). After incubation (1 h, RT) and centrifugation (10 min at 4000×g), the supernatant (crude cell extract) was loaded to a column for purification. The samples from the 50-ml cultivations were loaded on to a HisTrap FF Crude 1-ml column (GE Healthcare) equilibrated with 20 mM sodium phosphate, 0.5 M NaCl, 10 mM imidazole, pH 7.4. The column was washed with the equilibration buffer, and bound protein was eluted with a linear gradient from 10 to 500 mM imidazole. DERA containing fractions were pooled and the buffer was changed to 50 mM Tris-HCl pH 7.5 by EconoPac (BioRad) desalting columns. The protein purity was verified with SDS-PAGE.

The *Ec* DERA mutants were purified from the small-scale cultivations (3 ml and 10 ml) with 0.2-ml HisPur™ Ni-NTA Spin Columns (Thermo Scientific) according to manufacturer’s instructions and the buffer was exchanged with PD-10 (GE Healthcare) desalting columns. The protein concentrations were determined by measuring the absorbance at 280 nm and calculated using the theoretical epsilon based on the amino acid sequence (monomer).

### Cloning, protein expression and purification of Klebsiella pneumoniae 1,3-propanediol oxidoreductase (Kp PDOR)

*Klebsiella pneumoniae* 1,3-propanediol oxidoreductase (*Kp* PDOR; UniProt Q59477) encoding gene *dhaT* with a N-terminal 6× His-tag was codon optimised for *E. coli* and synthesised by Integrated DNA Technologies and cloned into the pBAT4 vector in a similar manner as described above for DERA. For the codon-optimised nucleotide sequence, see “GenBank accession numbers” section. Expression of *Kp* PDOR was done in *E. coli* BL21(DE3) by cultivating the *Kp* PDOR expression vector containing strain in 50-ml volume in 250-ml shake flasks in LB-medium (100 μg ampicillin/ml) similarly as for DERA described above. Purification of *Kp* PDOR was also done in the same way as for DERA. The purified fractions of *Kp* PDOR were pooled and the buffer was exchanged with EconoPac desalting columns to 50 mM Tris-HCl pH 7.5, 2 mM DTT, 1 mM MnCl_2_.

### Following DRP and DR cleavage reactions by DERA enzymes

The cleavage of the natural DERA substrate, deoxyribose 5-phosphate (DRP), was measured using 2-deoxyribose 5-phosphate sodium salt (Sigma-Aldrich) as a substrate in a coupled enzyme system with triosephosphate isomerase (TPI) and glycerol 3-phosphate dehydrogenase (GPD) from rabbit muscle (Sigma-Aldrich) in ambient temperature. DERA activity on DRP liberates glyceraldehyde-3-phosphate, which is reduced to glycerol-3-phosphate by the supplementary enzymes TPI and GDH. The latter reaction consumes NADH, which can be detected by spectrophotometer. The reaction mixture contained 0.1 μM purified DERA wild-type or variant, 5 mM DRP, 3 units of TPI, 2 units of GPD and 0.3 mM NADH in 50 mM Tris-HCl, pH 7.5, supplemented with 5 mM MgCl_2_. The reaction was initiated by addition of DRP and followed by measuring the decrease of absorbance at 340 nm using a Varioskan microtiter plate reader (Thermo). DERA activity on non-phosphorylated substrate 2-deoxy-D-ribose (DR, Sigma-Aldrich) was assayed similarly in a coupled enzyme system with 4 units of alcohol dehydrogenase (ADH) from *Saccharomyces cerevisiae* (Sigma-Aldrich) using 50 mM DR and 2 μM purified DERA wild-type enzyme or variant, and 0.3 mM NADH in 50 mM Tris-HCl, pH 7.5, supplemented with 5 mM MgCl_2_. Cleavage of DR by DERA liberates acetaldehyde, which is converted to ethanol by ADH in NADH consuming reaction.

### Sequential aldol addition reaction of acetaldehyde by DERA enzymes

The sequential aldol addition of acetaldehyde was monitored by incubating 5 μM DERA with different amounts (10–50 mM) of acetaldehyde in 50 mM Tris-HCl buffer pH 7.5 in ambient temperature for 20 h. The reactions were stopped by addition of acetonitrile (20 μl of reaction mixture + 80 μl of acetonitrile), clarified by centrifugation and analysed with a UPLC system (Waters) equipped with photodiode array detector. An Acquity BEH Amide column (2.1 × 100 mm, 1.7 μm, Waters) was used in 40 °C with 0.6 ml/min flow rate. The solvents used in the UPLC were eluent A: 50% acetonitrile/50% H_2_O and 10 mM ammonium acetate pH 9 and eluent B: 95% acetonitrile/5% H_2_O and 10 mM ammonium acetate pH 9. The column was equilibrated with 99.9% B, and 5 μl of sample was injected and eluted with program as follows: 0–0.4 min isocratic 99.9% B, 0.4–0.5 min gradient to 60% B, 0.5–2.0 min gradient 30% B and 2–5 min isocratic 99.9% B. The acetaldehyde concentration was followed by adsorption at 285 nm, and the formation of the aldol addition product by adsorption at 217 nm.

### Aldol addition of formaldehyde and acetaldehyde by DERA enzymes

The addition reaction of formaldehyde and acetaldehyde was monitored by incubating 5 μM DERA with 2 mM formaldehyde and 2 mM acetaldehyde in 50 mM Tris-HCl buffer pH 7.5 in ambient temperature. The reaction was stopped by addition of 2,4,-dinitrophenylhydrazine (2,4-DNPH) and acetonitrile, which also initiated the derivatisation reaction (Allen 1930). For derivatisation, typically 25 μl of the DERA reaction mixture was transferred to an Eppendorf tube containing 70 μl of 2,4-DNPH mixture (5 μl of saturated 2,4-DNPH, 65 μl acetonitrile, and 30 mM phosphoric acid). The derivatisation reaction was allowed to proceed at 22 °C for 1 h or overnight in + 4 °C. After derivatisation, the samples were clarified by centrifugation and injected to an Acquity BEH UPLC C18 column (2.1 mm × 50 mm, 1.7 μm, Waters) equilibrated with 70% H_2_O, 30% acetonitrile and eluted with isocratic elution with the equilibration buffer using 0.5 ml/min flow rate. The derivatised aldehydes were detected by measuring the absorbance at 360 nm (Allen 1930).

### Identification of the aldol addition product of formaldehyde and acetaldehyde

Propanediol oxidoreductase is an NAD-dependent enzyme that oxidises 1,3-propanediol (1,3-PD) to generate 3-hydroxypropionaldehyde (3-HPA). *Kp* PDOR was incubated with 30 mM 1,3-PD, 5 mM NAD, in 50 mM Tris-HCl buffer, containing 2 mM DTT and 1 mM MnCl_2_, pH 7.5 in ambient temperature. The aldehydes in the reaction were detected by reversed-phase UPLC after derivatisation with 2,4-DNPH as described above.

The mass of the product of DERA catalysed addition reaction of 10 mM formaldehyde and 10 mM acetaldehyde after 2-h incubation at 22 °C was analysed by LC-MS using a C18 column. After derivatisation with 2,4-DNPH, the reaction was separated using 75:25 water plus 1% formic acid/acetonitrile.

### Analysis of DERA catalysed aldol addition products by NMR spectroscopy

NMR experiments were carried out at 22 °C in 50 mM Na-phosphate buffer, pH 6.8, containing 10% of D_2_O (Aldrich). Bruker Avance III NMR spectrometer equipped with a QCI H-P/C/N-D cryoprobe was used. In 1D ^1^H experiments, the water signal was suppressed by 4-s-long volume selective presaturation (so-called NOESY presaturation) using Bruker’s pulse program *noesygppr1D*. For 2D COSY, TOCSY, HSQC and HMBC standard Bruker pulse programs with water signal presaturation were used. In TOCSY, the mixing time (DIPSI2) was 80 or 120 ms, and in HSQC, adiabatic inversion pulses were used and the ^1^H decoupling was achieved by adiabatic CHIRP sequence. The long range ^1^H,^13^C coupling constant in HMBC was set to 8 Hz. The chemical shifts were referenced to internal TSP (3-propionic-2,2,3,3-d4 acid sodium salt, Aldrich). The spectra were processed with Topspin 3.5, pl 7 software (Bruker).

### Circular dichroism spectroscopy to determine the thermostability

Temperature-induced unfolding of the purified DERA proteins from different organisms was measured by circular dichroism (CD) spectroscopy. The purified DERAs were diluted in 10 mM Tris-HCl buffer, pH 7.5 to 3 μM concentration. CD spectra were recorded from 240 to 190 nm using a 1 mm cell and a bandwidth of 1 nm with Chirascan CD spectrophotometer (Applied Photophysics, UK) at 20 °C. The unfolding curves were measured at 222 nm by gradually increasing the sample temperature with a gradient of 2 °C/min until a temperature of 90 °C was reached.

### Development of machine learning (ML) models for DERA mutant screening

**Fig. 1:**
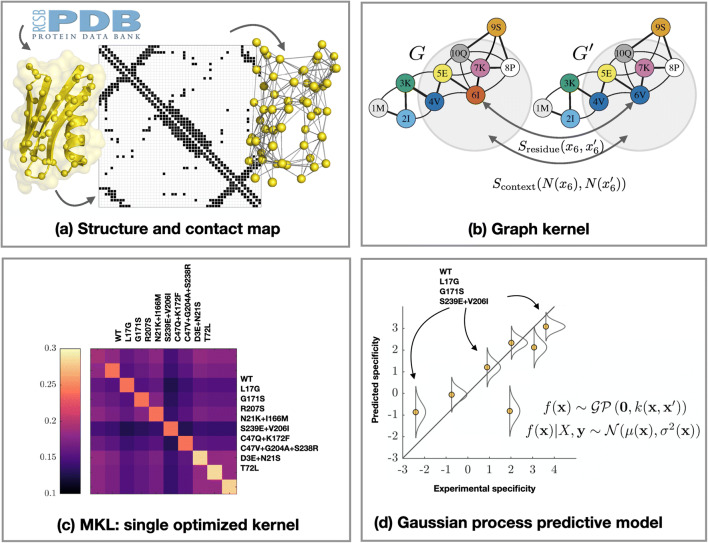
Illustration on the machine learning (ML) framework used in this work. The DERA protein structure is encoded as a contact map (**a**), which is combined with multiple node and edge substitution matrices to compute a graph kernel (**b**). We performed multiple kernel learning (MKL) (**c**) to find an optimised kernel to be used in Gaussian process predictive model (**d**). The kernel matrix measures variant similarities informative for substrate specificity. The substrate specificity predictive model is trained using experimental data

A novel ML model was used to automatically predict substrate specificities of DERA mutants based on Gaussian processes, as summarised in Fig. 1. See the Supplementary material Text S1 for a more detailed description of the ML model.

### X-ray crystallography of Ec DERA variants

The three-dimensional structures for two *Ec* DERA variants were determined by X-ray crystallography. The N21K mutant, as a 1.2-mg/ml solution in 50 mM Tris-HCl buffer, pH 8.0, could be crystallised directly from the buffer. The C47V/G204A/S239D mutant, as 1.3-mg/ml solution in 50 mM sodium phosphate buffer, pH 7.5, precipitated immediately in all conditions, and therefore, it was transferred to 50 mM Tris-HCl, pH 8.0 buffer for crystallisation using a PD-10 desalting column and concentrated to 3.2 mg/ml. Concentration was done in a centrifuge using a Vivaspin 2 column with 10 kDa molecular weight cut-off.

Both protein samples were crystallised using hanging-drop vapour diffusion. The N21K mutant was crystallised directly from a crystallisation solution with 18–20% PEG4000, 0.2 M magnesium formate and 0.1 M Tris-HCl pH 8.0. The C47V/G204A/S239D mutant formed large, rugged needles in 21% PEG3350, 0.2 M magnesium formate and 0.1 M Bis-Tris, pH 6.5, which were crushed and used in streak seeding, which produced good crystals in the same conditions but 17–18% PEG3350. Prior to X-ray diffraction measurements, crystals were soaked in solutions equivalent to their respective crystallisation solutions plus 0.1 M ligand, D-2-deoxyribose-5-phosphate (DRP), for 2 days.

Crystals were measured at European synchrotrons. They were mounted in nylon loops in cryoprotectant solutions equivalent to their respective crystallisation solutions but with 40% PEG, stored in liquid nitrogen and sent to the synchrotrons for remote measurement. Crystals of the N21K mutant were measured on the ID30A-1 beamline at the European Synchrotron Radiation Facility (ESRF), and crystals of the C47V/G204A/S239D mutant were measured on the I24 beamline at Diamond Light Source (DLS). Autoprocessed MTZ files generated by EDNA_proc and xia2-3dii programs were selected for structure determination for crystals measured at ESRF and DLS, respectively.

In addition, a large crystal of the N21K mutant was measured using the home X-ray diffractometer: Nonius FR591 rotating anode X-ray source by Bruker, mar345dtb goniometer system and mar345 image plate detector by X-ray Research (marXperts). The crystal was transferred to a cryoprotectant solution with 40% PEG4000, 0.2 M magnesium formate and 0.1 M Tris-HCl, pH 8.0 and placed into the sample holder in a nylon loop, where it was cooled down to constant 100 K in cold nitrogen stream. The detector was set to the minimum allowed distance of 150 mm, equivalent to 1.86 Å resolution limit. A data set was collected, and the crystal diffracted beyond the resolution limit. Images were processed with XDS program package (Version November 1, 2016).

All structure determination calculations were done with PHENIX software suite (Moriarty et al. 2009; Chen et al. 2010; Adams et al. 2010). Phasing was done using phenix.phaser (McCoy et al. 2007) molecular replacement, and previously published wild-type DERA structure (PDB entry 1KTN, no article published) was used as initial model. Mutations were done manually in Coot (Emsley et al. 2010) after molecular replacement. Structures were then refined using phenix.refine (Afonine et al. 2012). Water molecules were first added using the “Update waters” option and later checked and corrected manually. Appropriate ligands were placed in the active sites, and the aldehydes bound as Schiff base were connected to the amino group of the catalytic lysine with appropriate geometry restraints. Presence of partial DRP in the N21K mutant structures was further confirmed by calculating Polder maps (Liebschner et al. 2017). For the final refinement rounds, weight optimisation options were enabled.

### GenBank accession numbers

The nucleotide sequences of codon-optimised DERA and PDOR-coding genes used in this study can be found in the GenBank with the following accession numbers: MT702750 for DERA-coding gene from *E. coli*, MT702753 for DERA-coding gene from *Geobacillus*, MT702754 for DERA-coding gene from *Thermus thermophilus*, MT702748 for DERA-coding gene from *Coccidioides immitis*, MT702749 for DERA-coding gene from *Lactobacillus brevis*, MT702752 for DERA-coding gene from *Talaromyces islandicus*, MT702751 for DERA-coding gene from *Aspergillus niger* and MT682136 for *Klebsiella pneumoniae dhaT* gene.

## Results

### Selecting the most suitable DERA enzyme for the protein engineering work

Several known DERA enzymes of bacterial and fungal origin were initially considered as a target enzyme for the protein engineering work. For the proper comparison, we decided to express in *E. coli* the DERA aldolases from *E. coli*, *Geobacillus* sp., *Thermus thermophilus*, *Lactobacillus brevis*, *Coccidioides immitis*, *Aspergillus niger* and *Talaromyces islandicus*. The characterisation data of the purified enzymes is shown in Table 1. All seven DERAs were shown to be promiscuous, accepting both the natural substrate DRP and the non-phosphorylated version, DR. Moreover, acetaldehyde was also shown to be an acceptor substrate for all the characterised DERAs (as measured in the addition reaction). As high expression level and good thermostability were also considered to be relevant properties, *E. coli (Ec)* DERA was chosen as the target for our mutagenesis work.

**Table 1:** Characterisation of seven purified DERA enzymes, expressed in *E. coli* and purified with a His-tag

Microbial source of the DERA enzyme	Yield of purified protein from 50-ml cultivation	Relative activity on 5 mM DRP^a^	Relative activity on 50 mM DR^a^	Relative activity on 30 mM acetaldehyde^a^	*T*_m_ (°C)^b^
*E. coli*	12 mg	1	1	1	65 ± 1
*Aspergillus niger*	2.1 mg	0.3	1.0	0.4	48 ± 1
*Talaromyces islandicus*	2.5 mg	0.3	0.6	0.9	47 ± 1
*Geobacillus* sp.	3.8 mg	0.4	1.4	1.4	75 ± 1
*Thermus thermophilus*	~ 1 mg	0.1	0.7	0.7	≥ 90
*Coccidioides immitis*	1.4 mg	0.3	0.8	1.1	39 ± 1
*Lactobacillus brevis*	1.7 mg	0.6	0.3	1.2	38 ± 1

*DRP* deoxyribose-5-phosphate, *DR* deoxyribose

^a^Activities are presented relative to *E. coli* DERA activities

^b^Thermostability (*T*_m_) of the purified protein was determined with CD spectroscopy

### Setting up the analytics for the DERA-catalysed reactions

The activity measurements for DERA wild-type and mutants in the cleavage direction, e.g. cleavage of the natural substrate DRP and the non-phosphorylated substrate DR, were carried out based on the methodology described in the literature. These methods are applicable also with crude cell extracts (data not shown), but we decided to purify the enzymes to be able to accurately compare the specific activities of the different DERA variants (including different DERAs and *Ec* DERA mutants).

The assay for acetaldehyde addition activity was set up for LC using an amide column in alkaline conditions. It was noticed that during the course of the DERA reaction, a product peak appeared (Fig. S1), which could be detected by absorption at 217 nm. The main product of DERA activity on acetaldehyde has been shown to be an aldol addition product of three acetaldehyde molecules in a sequential reaction, where DERA first adds two acetaldehydes to form an C4 aldehyde (3-hydroxybutanal), which is then once more coupled with acetaldehyde into a C6 product, 2,4,6-trideoxyhexose. This product cyclises spontaneously to a hemiacetal and is thus removed from the reaction (Gijsen and Wong 1994). The C6 product formation from the DERA-catalysed reactions could not be quantified in the LC assay due to the fact that 2,4,6-trideoxyhexose is not commercially available as a standard. However, the identification of the formed cyclic trideoxyhexose product was verified by NMR. In the NMR experiments, several products from the enzymatic reaction were observed. After identifying the different spin systems from TOCSY spectra, their structures were determined by standard 2D NMR methods and the chemical shifts were compared with those published in Dick et al. (2016). The main products were the first aldol addition product 3-hydroxybutanal (17%) and the second aldol addition product that has undergone a spontaneous cyclisation to two anomers of pyranose rings (73% and 10%). In addition, a very small amount of crotonaldehyde was detected, a condensation product from two acetaldehyde molecules, that has been reported to be a side-reaction product of DERA (Dick et al. 2016).

Acetaldehyde addition reaction was carried out using acetaldehyde as a sole substrate, i.e. acetaldehyde acts both as acceptor and donor substrate. In the literature, very high acetaldehyde concentrations (e.g. 300–500 mM) are often used to demonstrate the synthesis of 2,4,6-trideoxyhexose with DERA. However, high acetaldehyde concentrations have also been shown to inhibit the DERA activity (Jennewein et al. 2006; Dick et al. 2016; Bramski et al. 2017). The conditions for the enzymatic acetaldehyde addition reaction were thus chosen so that roughly saturating substrate concentration of 30 mM was used. The acetaldehyde standards were reproducible in our LC method; however, the acetaldehyde concentrations in the presence of DERA, measured before or after incubation, were noisier. This was assumed to be because of covalent binding of the acetaldehyde to the enzyme protein (Dick et al. 2016).

In order to analyse DERA-catalysed addition reaction of acetaldehyde with another aldehyde, activity assay based on derivatisation of the aldehydes with 2,4-DNPH in acidic conditions, followed by a reversed-phase LC separation with UV detection, was set up. This method allowed detection of the aldehyde substrates as well as formed aldol addition products present in the DERA reactions.

### About the DERA protein engineering approaches in this work

Engineering of the substrate specificity is often attempted through rational mutagenesis, targeted near the active site or the substrate binding area. Even though the 3D structure of *Ec* DERA is available in high resolution and also in complex with the natural substrate DRP (Heine et al. 2001), it was challenging to rationally design mutations towards improved activity on small non-phosphorylated aldehydes. On the other hand, directed evolution including random mutagenesis to the whole gene, or even random mutagenesis to a targeted area, puts a stress on screening of the activities in high throughput manner. In the present study, we used targeted mutagenesis during the 1st round to create single amino acid mutants of *Ec* DERA. The most beneficial mutants were then combined based on activity data to create double and triple mutants. Furthermore, saturation mutagenesis at certain amino acid spots was also carried out. Finally, during the 3rd round, double or triple point mutants were created using machine learning algorithm predictions, explained in more details in Supplementary section and below. Altogether, roughly 150 *Ec* DERA mutants were characterised during the course of the work.

### Site-directed mutagenesis to make single amino acid mutations to Ec DERA (1st round)

**Fig. 2:**
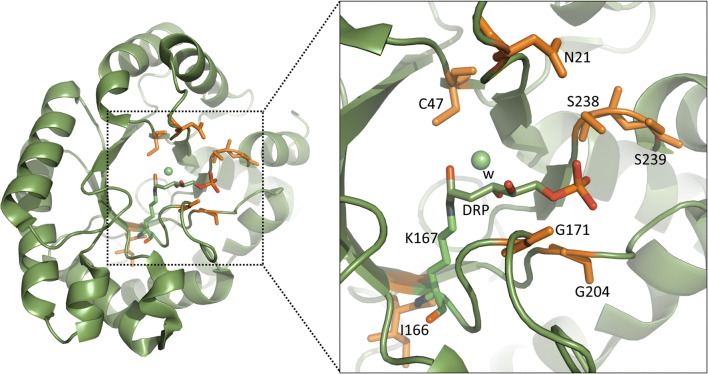
The 3D structure of covalent complex of *E. coli* DERA with DRP (PDB id. 1JCL Heine et al. 2001). Mechanistically relevant water molecule is shown as light-blue sphere. Residues N21, C47, I166, G171, G204, S238 and S239, which had the most beneficial effects, mutated in the study are shown in orange. The Schiff-base forming lysine (K167) is shown in green

**Fig. 3:**
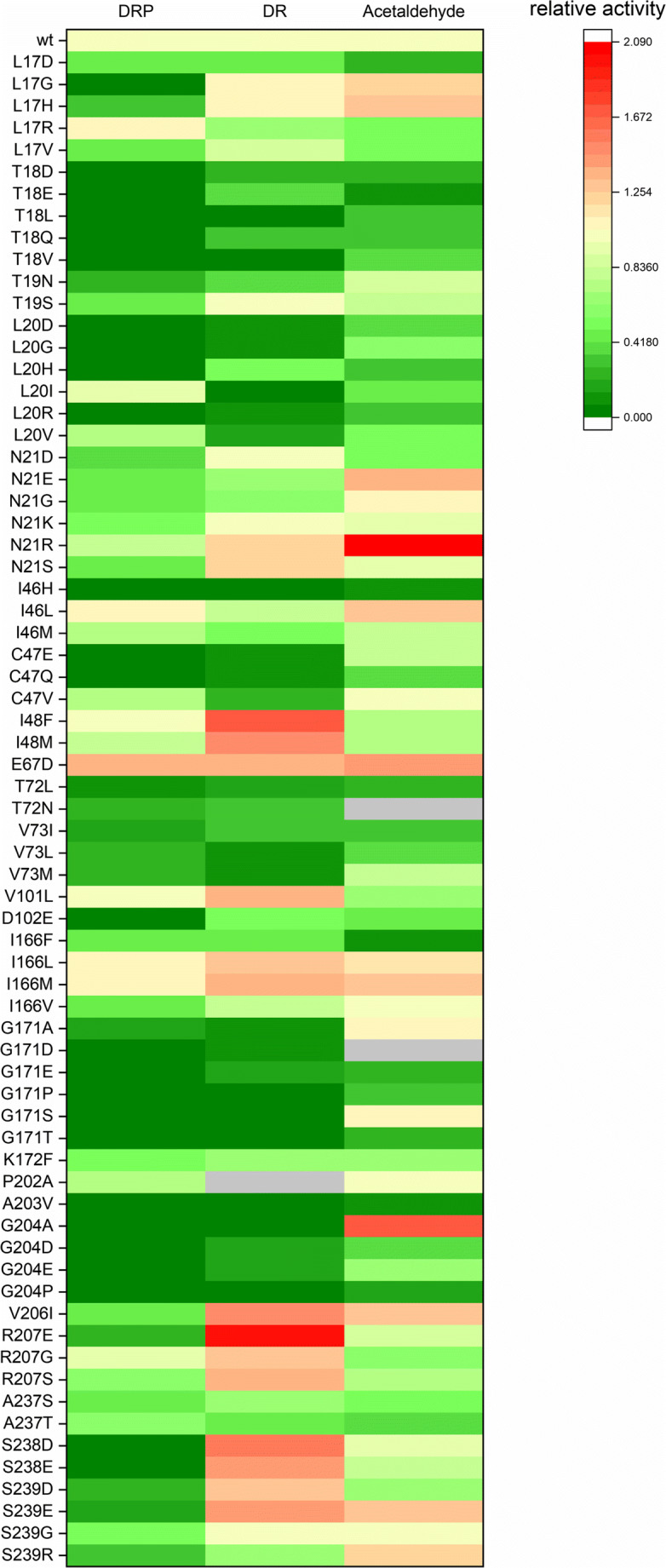
Heatmap displaying the 2-deoxyribose 5-phosphate (DRP) and 2-deoxyribose (DR) cleaving activities and acetaldehyde addition activities of all *Ec* DERA single point relative to the wild-type enzyme (wt)

**Fig. 4:**
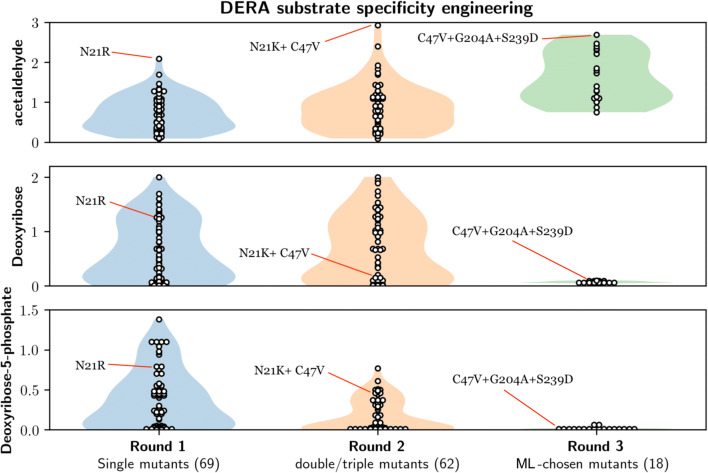
Summary of the substrate specificities of *Ec* DERA variants on deoxyribose-5-phosphate (DRP), deoxyribose (DR) and acetaldehyde (rows) over three mutagenesis rounds (columns), demonstrating that the ML-optimised mutants have abolished activities on DRP and DR, and increased acetaldehyde specificity. The white circles indicate specificities of individual DERA variants, and the shaded violin plots indicate smoothed vertical histograms (i.e. overall number as percentages from the total number of mutants)

Altogether, 69 single *Ec* DERA mutants, targeting 24 amino acid positions, were created with site-directed mutagenesis, expressed in *E. coli* as His-tagged proteins, and purified with Ni-NTA spin columns. The amino acid positions to be mutagenised were chosen mainly by examining the high-resolution crystal structure in complex with the native *Ec* DERA substrate, DRP (Heine et al. 2001; PDB id. 1JCL). In addition, sequence alignments of DERAs from different origins were utilised to find conserved amino acids and possible targets for consensus mutations. Moreover, the online tool HotSpot-Wizard (Bendl et al. 2016) and literature were utilised when selecting the residues to be mutated. Most of the mutations were targeted in close vicinity (maximum 4 Å distance) of the active site and the substrate binding pocket (Fig. 2). The activities of the purified *Ec* DERA variants were measured with three different substrates: DRP and DR for the cleavage reaction and acetaldehyde for the aldol addition reaction. Altogether, 30 variants having single-point mutations had clearly reduced specific activity (20% or less) towards the natural substrate DRP as compared with the wild-type enzyme (Figs. 3 and 4 and S2). Of these 30 *Ec* DERA variants, five showed additionally improved activity on acetaldehyde aldol addition, i.e. the *Ec* DERA mutants G204A, S239E, L17G, G171A and G171S (Fig. S2).

### Ec DERA variants containing two or three amino acid mutations (2nd round)

Altogether, 62 double or triple *Ec* DERA mutants were created by (a) manually combining the most interesting single mutations and (b) by using saturation mutagenesis on selected spots, as described in more details under Materials and methods section. In the manual combination, one of the selection criteria used was to pick mutations that affected the acetaldehyde addition activity. The *Ec* DERA variants included 34 double or triple mutants, which were combined from the single mutants based on the activity data. In addition, 28 double mutants were created using saturation mutagenesis on amino acids C47, I166 and S238. In each case, these mutants were made on top of the *Ec* DERA variant N21K. The rational in picking the spots for the saturation mutagenesis was that these three amino acid positions C47, I166 and S238 were found to affect favourably to the acetaldehyde addition reaction (Fig. 3). Moreover, C47 has been previously shown to be a target for inactivation by aldehydes (Dick et al. 2016) and based on the *Ec* DERA wild-type complex structure. In addition, S238 is hydrogen bonded to the phosphate group of the natural substrate DRP. Overall, several interesting variants having low activity on both DRP and DR substrates and improved acetaldehyde addition activity were discovered among all the screened double and triple *Ec* DERA variants in the 2nd mutagenesis round, e.g. N21K/C47V, N21K/C47L, N21K/C47F, N21K/C47S, and N21K/S238G. Additionally, the *Ec* DERA variants S239/V206I/L17H, S239/V206I/I166M, and S239D/V206I showed reduced activity on DRP and improved acetaldehyde addition activity (Figs. 4 and S3). It should be also noted that in most cases, no additivity effect of point mutations in terms of activity data could be detected.

### Ec DERA variants created using machine learning (3rd round)

A novel machine learning (ML) model to automatically predict substrate specificities of DERA mutants based on Gaussian processes was developed. Our goals were to train the specificity prediction functions from specificity observations and use these subsequently for screening new potential *Ec* DERA variants. See the Supplementary material Text S1 for a technical description of the development and training of the ML model.

A machine learning model was trained using the data from the available *Ec* DERA mutants, consisting of (i) the 69 single point mutants and (ii) the 62 double or triple point mutants, in total 131 mutants. Each mutant had measured data on DRP, DR and acetaldehyde specificity. The ML model was trained to predict all three substrate specificities. In Fig. S4, the trained combinations of substitution models are shown. The DRP model ended up using six different substitution models whereas the DR model used five, with amino acid interaction features having most predictive information. The acetaldehyde model only used four substitution models with contact energy and packing features having highest weights.

The predicted and measured activity data of the *Ec* DERA mutants are shown in Fig. S5. The ML model achieved cross-validation test correlation on the first two rounds of 0.57 for DRP, 0.81 for DR and 0.54 for acetaldehyde, respectively. The 131 data points were sufficient for the ML model to explain the substrate specificity of each of them.

After this, we screened in silico all possible *Ec* DERA variants with 1–3 amino acid mutations using the ML model, in total 48,000 new variants. These were sorted based on the predicted acetaldehyde specificity, subtracted with DRP specificity to find the most probable candidates with high acetaldehyde but low DRP specificity. From the top 50 best estimated variants, 18 mutants were manually chosen for in vitro mutagenesis experiments.

The results of these 18 *Ec* DERA variants (containing two or three point mutations) showed that all except three had improved activity on acetaldehyde addition, and five of the variants had more than two-fold improved acetaldehyde addition activity (Figs. 4 and 6). In addition, for all 18 *Ec* DERA mutants, the DR and DRP activity was almost completely abolished (Figs. 4 and 6). The Fig. S6 shows the correlations of the 18 final variants, which indicate a 0.99 correlation for DRP and DR due to their successful specificity removal and a 0.96 correlation between estimated and measured acetaldehyde specificity. We note that one should not directly compare these correlations to the cross-validation correlations due to active selection procedure of the 3rd round variant.

### Crystal structure analysis

The crystal structures of two variants of *Ec* DERA were determined, in order to elucidate mutation-induced changes in the 3D protein structure. The crystal structure of *Ec* DERA N21K variant, which had lowered specific activity on DRP, and wild-type like activity on DR and acetaldehyde, was determined with and without the ligand, D-2-deoxyribose-5-phosphate (DRP). In addition, the crystal structure of one of the best *Ec* DERA variants, C47V/G204A/S239D, having improved activity towards acetaldehyde and basically no activity on DRP or DR, derived from the 3rd mutagenesis around, was determined from the crystal soaked with DRP. Diffraction resolutions for all three structures were high (1.5 to 1.9 Å) and the crystallographic R-factors were very low (Table S2), indicative of high quality 3D structures in each case. The determined three 3D structures were superimposed with the crystal structures of the *Ec* DERA wild-type without (pdb code 1p1x, 1.0 Å resolution) and with a ligand complex (1-hydroxy-pentane-3,4-diol-5-phosphate)(1jcl, 1.1 Å resolution), to analyze mutant-induced changes in the 3D structures.

**Fig. 5:**
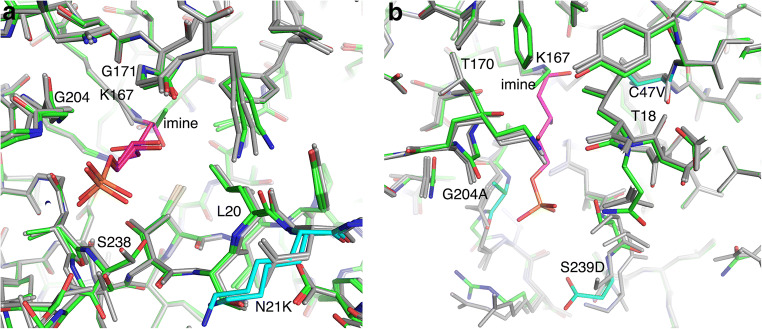
The superimposition of the crystal structures of the *Ec* DERA wild-type and two variants around the active site as stick models. *Ec* DERA wild-type structures as uncomplexed and complexed form are shown in grey. The covalently bound 1-hydroxy-pentane-3,4-diol-5-phosphate is in purple. **a** The superimposition with the uncomplexed and complexed form of *Ec* DERA N21K variant. Variant structures in uncomplexed and complexed forms are shown in green and bound ligand, glyceraldehyde-3-phosphate, in purple. **b** The superimposition with the uncomplexed form of *Ec* DERA C47V/G204A/S239D variant. The variant structure is shown in green and the mutated residues are in cyan.

The complex structure of *Ec* DERA N21K mutant revealed binding of the reaction product from DRP cleavage, i.e. glyceraldehyde-3-phosphate (C3P), to the acceptor site in one of the two protein molecules in the asymmetric unit (Fig. 5a). A similar conformational change in the loop around residue S238 in both the Ec DERA N21K mutant and wild-type structures can be seen upon glyceraldehyde-3-phosphate binding, when uncomplexed and complexed crystal structures are compared. In addition, in both N21K crystal structures, there are slight (about 0.2 to 0.4 Å) movements in the positions of altogether three active site loops containing residues G171, G204 and L20, respectively, when compared with the wild-type DERA crystal structure. The N21K mutation is located in the third loop. These small movements slightly narrow the access to the active site of the N21K variant and may thus contribute to the decreased activity against DRP in many of the N21K containing double mutants tested in this work.

The structure of *Ec* DERA C47V/G204A/S239D variant was determined from crystals after soaking with DRP. The crystal structure showed a presence of a very small covalent adduct to the catalytic amino acid K167 in both molecules in the asymmetric unit. The electron density map showed elongated electron density corresponding to approximately two carbon atoms. In the refinement, it was modelled as an imine group representing a reaction intermediate. No binding of DRP or glyceraldehyde-3-phosphate could be detected. This could imply that binding of the DRP is clearly diminished in the C47V/G204A/S239D variant (Fig. 5b). The conformational differences in this variant as compared with the *Ec* DERA wild-type structure are clearly detectable. The conformation of the loop containing mutation G204A has pushed the position of its Cα atom by 1.0 Å towards active site, thus narrowing it. In addition, there are clear changes in the loop structure containing the S239D mutation. Because the residues of this loop participate in phosphate binding (of glyceraldehyde-3-phosphate acceptor), the S239D mutation would probably decrease binding of DRP both by introducing a negatively charged amino acid residue (phosphate being also negatively charged) and by narrowing the active site entrance. On the other hand, the C47V mutation, located relatively close to the catalytic K167 residue (about 5 Å), has caused only minimal alteration in the position of this amino acid residue, as it is located in the middle of β-strand.

### Further substrate promiscuity testing of Ec DERA variants

The goal of the protein engineering work was to improve the substrate specificity towards smaller, non-phosphorylated aldehydes over glyceraldehyde-3-phosphate. Acetaldehyde is a two-carbon aldehyde, and the only shorter aldehyde is formaldehyde, which is the simplest existing aldehyde. We decided to test also formaldehyde as acceptor in the DERA reaction catalysed by *Ec* DERA variants. The reference test with the wild-type *Ec* DERA enzyme indicated that this was already able to add formaldehyde to the acetaldehyde donor. The verification of the aldehyde addition reaction was carried out using the reversed-phase chromatography after derivatisation with 2,4-DNPH. Here, the product by *Ec* DERA catalysis was found to elute with an identical retention time to the product of *Kp* PDOR catalysed oxidation of 1,3-propanediol (Fig. S7), which is known to be 3-hydroxypropionaldehyde (3-HPA) (Johnson and Lin 1987). Furthermore, the mass m/z 255.0729, which is identical to 2,4-DNPH derivatised 3-HPA, was detected in the reaction, also suggesting 3-HPA to be formed by *Ec* DERA from formaldehyde and acetaldehyde. Moreover, the DERA products of formaldehyde with acetaldehyde were identified as 3-hydroxypropionaldehyde (30%) and the corresponding hydrate (70%) by 1D and 2D NMR experiments. The ^1^H NMR spectrum of the products and the product structures are shown in Fig. S8. Interestingly, the DERA addition reaction products of acetaldehyde with itself were not observed here at all.

After this, altogether, 140 *Ec* DERA variants were assayed for addition activity on formaldehyde and acetaldehyde. Of these, six *Ec* DERA variants were found to have improved activity as compared with the wild-type enzyme (Fig. S9). Two of the mutants had single mutations, two were double and two triple mutants. The relatively small number of *Ec* DERA variants with higher activity on formaldehyde addition to acetaldehyde as compared with the wild-type enzyme was not surprising, as both the screening and the machine learning prediction algorithm were set up to maximise the acetaldehyde addition reaction.

## Discussion

Deoxyribose-5-phosphate aldolases (DERAs) are acetaldehyde-dependent, Class I aldolases catalysing in nature a reversible aldol reaction between an acetaldehyde donor (C2 compound) and glyceraldehyde-3-phosphate acceptor (C3 compound, C3P) to generate deoxyribose-5-phosphate, DRP (C5 compound). The interesting feature is the substrate promiscuity as DERA enzymes have been shown to accept a wide range of aldehydes as acceptor molecules, thus offering a biocatalytic alternative for a (stereo)selective synthesis of C–C bonds. Substrate specificity on the donor substrate side is stricter but recent biodiversity screen has revealed that some DERAs also display nucleophile substrate promiscuity (Hernández et al. 2018; Chambre et al. 2019). Furthermore, DERA enzymes can carry out a tandem reaction with acetaldehyde as a sole substrate, leading to formation of a C6 product, 2,4,6-trideoxyhexose, which cyclises spontaneously and is removed from the reaction. DERA enzymes have been utilised in large-scale synthesis of pharmaceutical intermediates, i.e. statin precursors and pyranoid building blocks, as well as preparation of different types of deoxysugars, deoxy-ketoses and deoxy-sialic acid (Haridas et al. 2018). Despite of being promising enzymes for application purposes, there are still some challenges related to their usage. In particular, enzyme inactivation under synthesis conditions represents a major obstacles (Jennewein et al. 2006; Dick et al. 2016; Bramski et al. 2017), and thus, variants having improved substrate binding towards non-natural substrates and/or resistance towards high aldehyde concentrations are desired.

The 3D structures of several DERA enzymes have been solved, and there are also a few complex structures available. These have revealed that despite of relatively low sequence identity, all DERA enzymes have the ubiquitous TIM (α/β)_8_-barrel fold where the catalytic amino acids as well as other amino acid residues around the active site seem to be relatively well conserved (Heine et al. 2001; Haridas et al. 2018). The DERA reaction proceeds via Schiff base formation between an active site lysine residue (K167 in *Ec* DERA) and the donor acetaldehyde substrate. The active site of DERA is located in a deep binding cleft, where the donor acetaldehyde binds to the bottom of the cleft and the acceptor glyceraldehyde-3-phosphate to the upper part, near the cleft entrance. Binding of the substrates to the active site cleft is mediated through hydrogen bonds, either directly or through water molecules. Concerning the acceptor site, the hydrogen bonds are particularly directed to the phosphate group of the native substrate (glyceraldehyde-3-phosphate) (Heine et al. 2001; Heine et al. 2004). Based on the published *Ec* DERA complex structures, residues specific for phosphate-binding are the backbone amide groups of S238 and G205 and via a water molecule the residues G204, V206, S239 and G171. Residue K172 provides both a counter-charge and forms a hydrogen bond (via a water molecule) with the phosphate group (Heine et al. 2001).

In this work, we wanted to study the substrate specificity of DERA with various protein engineering approaches, including also machine learning methods. We set as our goal to improve the overall performance of DERA on utilising non-phosphorylated short aldehydes (C3 and C2). Initially, we characterised seven different purified DERA wild-type enzymes of bacterial and fungal origin and found that several of them were promiscuous and could also accept non-phosphorylated aldehydes as the acceptor substrate (Table 1). *E. coli* (*Ec*) DERA was chosen for the protein engineering work as it showed relatively good promiscuous activity towards the desired reactions and had high expression level and good thermostability (Table 1). Additionally, high-resolution complex structures of *Ec* DERA exist both with and without bound substrate (Heine et al. 2001; Heine et al. 2004), and some mutagenesis studies to change its substrate preference have also been carried out, thus providing a good starting point for semi-rational mutagenesis approaches.

**Fig. 6:**
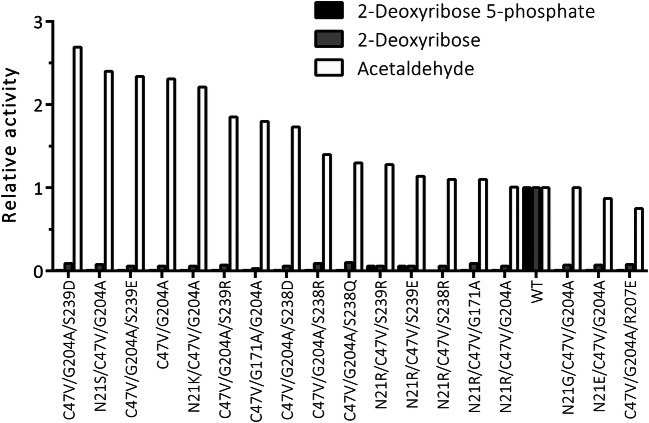
The characterisation data of the 18 *Ec* DERA variants containing two or three mutations, created by optimisation with machine learning algorithm. Cleaving activities on 2-deoxyribose 5-phosphate (DRP) and 2-deoxyribose (DR) and on acetaldehyde addition activity are shown as relative activities compared with the wild-type *Ec* DERA (WT)

DERA mutagenesis to alter the substrate specificity was targeted to the active site of the enzyme (Fig. 2). Initially, Web server HotSpot-Wizard, 3D structures of *Ec* DERA (Heine et al. 2001; PDB id. 1JCL) and literature were utilised when selecting the amino acid residues to be mutated. First mutagenesis round DERA variants were made as single amino acid mutants, and in the 2nd mutagenesis round, beneficial mutations were combined. Furthermore, saturation mutagenesis at certain amino acid spots was also carried out. The 3rd round of mutagenesis was carried out using machine learning (ML)-guided approach. The specific activities of the purified *Ec* DERA variants were measured initially with three different substrates: (1) DRP (cleavage reaction), (2) DR (cleavage reaction), (3) acetaldehyde (aldol addition reaction using acetaldehyde both as the donor and acceptor substrate). Altogether, roughly 150 purified *Ec* DERA mutants, having one to three point mutations, were characterised during the work. Several of the *Ec* DERA variants showed clear change in their substrate spectra (Figs. 3, 4, 6 and S4–S5). The most promising variants had substantially reduced, or completely abolished activity particularly towards the natural substrate (DRP), while showing activity on acetaldehyde addition reaction. Interestingly, we also discovered that most of the tested DERA wild-type enzymes could also accept, besides aldehyde, formaldehyde (C1 aldehyde) as the acceptor molecule. This prompted us to test the *Ec* DERA variants on formaldehyde (C1) utilisation (i.e. aldol addition reaction between acetaldehyde and formaldehyde). The results demonstrated that some of the mutants had also altered preference towards formaldehyde (C1 aldehyde), the following six variants being the most potent: L17H, N21D, G171T, N21R/R207E, V206I/S239R, N21R/D206I/S239R and E67D/N206I/S239R (Fig. S9). Interestingly, the acetaldehyde and formaldehyde activities did not always correlate, and none of the 3rd round variants (from the ML-guided mutagenesis) that had clear preference for acetaldehyde did not perform particularly well when formaldehyde was offered as an acceptor aldehyde. On the other hand, as discussed more thoroughly below, this also demonstrates the power of the ML-guided mutagenesis combined with screening.

During the work, a sample-efficient machine learning method for designing the optimal mutation strategy for protein engineering was developed. The proposed Gaussian process model is a principled statistical model that excels in moderate to low data settings. The model learns the posterior distribution of specificity functions from observations, while simultaneously performing feature learning for added interpretability of substitution model choices. The Gaussian process model excelled at interpolation and moderate extrapolation of the DERA structure. The results from the 3rd round of *Ec* DERA mutagenesis guided with machine learning (ML) algorithm aiming at high activity on acetaldehyde addition reaction, and low activity on DRP and DR substrates is shown in Fig. 6. As can be seen, all 18 variants tested had basically abolished activity towards the native DRP (i.e. glyceraldehyde-3P acceptor) as well as towards the non-phosphorylated DR substrate (i.e. toward glyceraldehyde acceptor). Moreover, 15 out of 18 variants tested had increased target specificity towards the C2 acceptor aldehyde. Thus, our ML model was able to successfully extrapolate from the characterisation data novel mutant combinations with desired specificity. The usage of the ML model enhanced the mutagenesis work by a dramatic improvement over the conventional mutant selection procedures and these type of methods are clearly useful to speed up the protein engineering work.

The five best *Ec* DERA variants having clearly improved substrate specificity towards acetaldehyde (Fig. 6) were (1) C47V/G204A/S239D, (2) N21S/C47E/G204A, (3) C47V/G204A/S239E, (4) C47V/G204A and (5) N21K/C47V/G204A. By examining all our mutant data, we conclude that mutations to the amino acid residues N21, C47, I166, G171, G204, S238 and S239 seemed to have the most beneficial effects on changing the substrate specificity (Figs. 3, 6, S2-S3). Most of these residues (i.e. G171, G204, S238 and S239, N21) are according to the structural data (by others and us, see also below) involved in binding the phosphate group of the glyceraldehyde-3-phosphate acceptor substrate. Residue I166 is located more distant to the active site, in the loop underlying the active site lysine residue K167. The reason for I166 mutations affecting to the substrate specificity is not clear; however, others have also noted that mutations to this spot affect the substrate binding (Jennewein et al. 2006). Furthermore, the conserved residue C47, which is located at the bottom of the active site cleft, seems to be able to form in some circumstances covalent adducts with aldehydes leading to enzyme inactivation (Dick et al. 2016; Bramski et al. 2017). All our best five mutants on acetaldehyde addition reaction contained a mutation C47V and it is plausible that this mutation promoted the enzymatic reaction by relieving the aldehyde inhibition. Interestingly also, the DERA from the hyperthermophile Archaea *Aeropyrum pernix* has naturally a Valine residue at this position (C47 in *Ec* DERA) (Sakuraba et al. 2003).

In order to further rationalise our protein engineering results, we determined the crystal structures of the two *Ec* DERA variants C47V/G204A/S239D and N21K as ligand complexes and compared these to the solved *Ec* DERA wild-type structures, as explained in more details under Results section (Fig. 5 and Table S2). We conclude that when aiming for improved binding of small non-phosphorylated aldehydes, mutations that narrow the substrate binding cleft near the entrance and/or affect to the binding of the substrate, in particular the phosphate group seem to be important. However, as evident from our variant data, purely rational design of the mutants remains challenging, and the substrate specificity improvement clearly benefited from saturation mutagenesis combined with ML-guided mutagenesis approaches. Overall, we could demonstrate that the synthetic utility of DERA enzyme may be substantially increased using protein engineering approaches.

## Supplementary Information

ESM 1 (PDF 1468 kb)
