# Supplementary material for: Substrate specificity of 2-deoxy-D-ribose 5-phosphate aldolase (DERA) assessed by different protein engineering and machine learning methods
Source: Appl Microbiol Biotechnol. 2020 Nov 4;104(24):10515–29. doi: 10.1007/s00253-020-10960-x (PMC7671976; doi:10.1007/s00253-020-10960-x)
Supplement: Supplementary file 1 — (PDF 1468 kb) [file 253_2020_10960_MOESM1_ESM.pdf]

AMB Applied Microbiology and Biotechnology

Substrate specificity of 2-Deoxy-D-ribose 5-phosphate aldolase (DERA) assessed by different protein engineering and machine learning methods

Sanni Voutilainen[1*], Markus Heinonen[2,3], Martina Andberg[1], Emmi Jokinen[2], Hannu Maaheimo[1], Johan Pääkkönen[4], Nina Hakulinen[4], Juha Rouvinen[4], Harri Lähdesmäki[2], Samuel Kaski[2,3], Juho Rousu[2,3], Merja Penttilä[1] and Anu Koivula[1]

Affiliations:

1 VTT Technical Research Centre of Finland Ltd, P.O. Box 1000, FI-02044 VTT, Finland

2 Department of Computer Science, Aalto University, Espoo, Finland

3 Helsinki Institute for Information Technology, Finland

4 Department of Chemistry, University of Eastern Finland, PO Box 111, FIN-80101 Joensuu, Finland

*To whom correspondence should be addressed:

Sanni Voutilainen

e-mail: sanni.voutilainen@vtt.fi

tel: +358405317015

SUPPLEMENTARY DATA

Table S1 Primers used for generation of the point mutants in the *Ec* DERA sequence. Nucleotide codes are according to IUPAC.

Text S1 Detailed description of the development of machine learning (ML) models for DERA mutant screening.

Fig S1 Setting up an LC assay for measuring the sequential acetaldehyde addition reaction by DERA. UPLC chromatogram was monitored at 217 nm for the product formation. Samples at time points 0 and 20 h containing either Ec DERA wild-type or BSA (as a negative control) are shown. This method was used to measure the acetaldehyde addition reaction of all the variants in this work.

Fig S2 *Ec* DERA single point mutations variants having clearly lowered activity on the natural substrate DRP (20% or less DRP activity left). The 2-deoxyribose 5-phosphate (DRP) and 2-deoxyribose (DR) cleaving activities and acetaldehyde addition activities of *Ec* DERA variants are shown as relative activities compared to the wild-type *Ec* DERA (WT).

Fig S3 2-deoxyribose 5-phosphate (DRP) and 2-deoxyribose (DR) cleaving activities and acetaldehyde addition activities of *Ec* DERA variants containing two or three mutations, combined based on activity data or made through saturation mutagenesis. The specific activities are shown as relative activities compared to the wild-type DERA (WT). The most interesting results from the mutants containing two or three mutations are shown.

Fig S4 The trained substitution matrix weights against the three substrate specificities of DERA variants (columns DRP, DR, C2), a higher value having more predictive information towards the specificity. Only 11 substitution models (rows) had non-negative weights. For DR specificity prediction, amino acid interactions was the most important feature, while contact energies were the most informative feature for acetaldehyde specificity prediction.

Fig S5 The 5-fold cross-validation test performance of the ML model on the characterization data from the first two mutagenesis rounds (altogether 131 *Ec* DERA mutants). The method achieves high correlation for DR specificity, and moderate correlation for DRP and acetaldehyde.

Table S2 X-ray data collection and refinement statistics for the *Ec* DERA variants.

Fig S6 The empirical accuracy of the ML-guided 18 Ec DERA variants from the 3rd mutagenesis round, which were predicted to have low activity on DRP and DR, and maximal activity on acetaldehyde. Due to the removal of the DRP and DR specificity, the predicted performance is high. The 18 *Ec* DERA variants estimated to have a high acetaldehyde specificity had a correlation of 0.956 with the observed specificities.

Fig S7 UPLC chromatogram at 360 nm for detection of the 2,4-DNPH derivatised products of DERA catalyzed addition of formaldehyde and acetaldehyde (blue line) and *Kp* PDOR catalysed oxidation of 1,3-propanediol (red line). The reaction products of both DERA activity and PDOR activity can be seen at around 0.6 min. Both DERA substrates, formaldehyde and acetaldehyde, are present after 20 h incubation, and they can be seen at around 1 min and 1.5 min elution time, respectively.

Fig S8 NMR identification of aldol addition product of formaldehyde and acetaldehyde by *Ec* DERA. 600 MHz ^{1}H NMR spectrum of the products 3-hydroxypropanal (A) and its hydrated form (B) of the DERA reaction of formaldehyde with acetaldehyde in 50 mM Na-phosphate buffer, pH 6.8, at 22°C. The assignments of the product signals to the ^{1}H atoms are indicated by the corresponding numbers.

Fig S9 The best *Ec* DERA variants on formaldehyde and acetaldehyde aldol addition reaction. Activities on four different assays, i.e. on 2-deoxyribose 5-phosphate (DRP) and 2-deoxyribose (DR) cleavage, acetaldehyde addition, and on formaldehyde + acetaldehyde addition activity are shown as relative activities compared to the wild-type *Ec* DERA (WT).

Table S3 The residue substitution matrices used in the model from AAindex database.

Table S4 The contact potential matrices used in the model from AAindex database.

Table S1 Primers used for generation of the point mutants in the *Ec* DERA sequence. Nucleotide codes are according to IUPAC.

Mutation	Primer (forward)	Primer (reverse)
L17G	GTTGATGGACggcACCACTCTGA	TTAAGCGCGCGAAGGCTAGA
L17H	GTTGATGGACcagACCACTCTGA	TTAAGCGCGCGAAGGCTAGA
T18E	GATGGACCTTgaaACTCTGAACG	AACTTAAGCGCGCGAAGGCT
T18L	GATGGACCTTctgACTCTGAACG	AACTTAAGCGCGCGAAGGCT
T18Q	GATGGACCTTcagACTCTGAACG	AACTTAAGCGCGCGAAGGCT
T19N	GGACCTTACCaacCTGAACGATGACG	ATCAACTTAAGCGCGCGA
T19S	GGACCTTACCagtCTGAACGATG	ATCAACTTAAGCGCGCGA
L20R, Q, E, H, V, D, G	GTTGATGGACSDNACCACTCTGAACG	TTAAGCGCGCGAAGGCTA
N21S, E, G, K, D, R	TACCACTCTGRRNGATGACGATAC	AGGTCCATCAACTTAAGC
C47V	GGCGGCCATTgtgATTTATCCGC	GTATTACCGACCGGCGTTTT
I46H	TACGGCGGCCcatTGCATTTATC	TTACCGACCGGCGTTTTT
I46L	TACGGCGGCCctgTGCATTTATC	TTACCGACCGGCGTTTTT
I46M	TACGGCGGCCatgTGCATTTATC	TTACCGACCGGCGTTTTT
I48F	GGCCATTTGCtttTATCCGCGTTTC	GCCGTATTACCGACCGGC
I48M	GGCCATTTGCatgTATCCGCGTTTC	GCCGTATTACCGACCGGC
T72L	CCGTATTGCGctgGTAACTAACTTCCCACATGGC	ATCTCCGGGGTGCCCTGT
T72N	CCGTATTGCGaacGTAACTAACTTCC	ATCTCCGGGGTGCCCTGT
V73I	TATTGCGACCattACTAACTTCCCACATGGC	CGGATCTCCGGGGTGCCC
V73L	TATTGCGACCctgACTAACTTCCCACATGGC	CGGATCTCCGGGGTGCCC
V73M	TATTGCGACCatgACTAACTTCCCACATGGC	CGGATCTCCGGGGTGCCC
V101L	TGCGGATGAGctgGACGTTGTATTTCC	CCATATGCGATTGCCGCC
D102E	GGATGAGGTCgaaGTTGTATTTC	GCACCATATGCGATTGCC
D102N	GGATGAGGTCaacGTTGTATTTCC	GCACCATATGCGATTGCC
V103M	TGAGGTCGACatgGTATTTCCGTATC	TCCGCACCATATGCGATTG
I166M	AGCGGATTTCatgAAAACGAGTAC	CCTGCTTTGATGGAGATC
I166V	AGCGGATTTCgttAAAACGAGTAC	CCTGCTTTGATGGAGATC
G171A	AACGAGTACGgcgAAAGTGGCTG	TTGATGAAATCCGCTCCTG
G171D	AACGAGTACGgatAAAGTGGCTG	TTGATGAAATCCGCTCCTG
G171E	AACGAGTACGgaaAAAGTGGCTG	TTGATGAAATCCGCTCCTG
G171P	AACGAGTACGccgAAAGTGGCTG	TTGATGAAATCCGCTCCTG
G171S	AACGAGTACGtctAAAGTGGCTG	TTGATGAAATCCGCTCCTG
G171T	AACGAGTACGaccAAAGTGGCTG	TTGATGAAATCCGCTCCTG
G204A	TAAGCCCGCCgcgGGTGTTCGCA	AAGCCTACCGTTTTTCAACAC

G204D	TAAGCCCGCCgatGGTGTTCGCA	AAGCCTACCGTTTTTTCAA CAC
G204E	TAAGCCCGCCgaaGGTGTTCGCA	AAGCCTACCGTTTTTTCAA CAC
V206I	CGCCGGCGGTattCGCACCGCGG	GGCTTAAAGCCTACCGTTT T
R207E, K, S, D, G, N, R	CGGCGGTGTTRRNACCGCGGAAG	GCGGGCTTAAAGCCTACC
A237S	TCGCTTTGGAagcAGTTCACTGCTGGC	TAATGGCGTGCATCAGCC
A237T	TCGCTTTGGAacgAGTTCACTGC	TAATGGCGTGCATCAGCC
S238D	CTTTGGAGCGgatTCACTGCTGGC	CGATAATGGCGTGCATCAG
S238E	CTTTGGAGCGgaaTCACTGCTGGC	CGATAATGGCGTGCATCAG
S238Q	CTTTGGAGCGcagTCACTGCTGGC	CGATAATGGCGTGCATCAG
S238R	CTTTGGAGCGcgtTCACTGCTGGC	CGATAATGGCGTGCATCAG
S239E, G, R, K, N, D	TGGAGCGAGTRRNCTGCTGGCTT	AAGCGATAATGGCGTGCAT C

Text S1 Detailed description of the development of machine learning (ML) models for DERA mutant screening.

Machine learning (ML) models were trained to automatically predict substrate specificities of DERA mutants based on Gaussian processes.

Gaussian processes

We used a Gaussian process (GP) prior for function f to predict the substrate specificities $f(x) \in R$ of a DERA protein mutant variant x. Each specificity was modeled as a separate function. Gaussian processes are a family of non-parametric, non-linear Bayesian models (Rasmussen and Willams 2006). A GP is a probabilistic model, where the prediction is represented as a Gaussian distribution with expectation $E[f(x)]$, variance $var[f(x)]$, and covariances $cov[f(x), f(x')]$, which are governed by the GP equations. We begin by defining a zero-mean GP prior

$$f(x) \sim GP(m(x), k(x, x')),$$

which defines a distribution over specificity functions $f(x)$ whose mean and covariance are $E[f(x)] = m(x)$ and $cov[f(x), f(x')] = k(x, x')$.

Without loss of generality, we assumed a zero-mean prior with $m(x) = 0$. For any collection of protein variants $X = (x_1, \dots, x_N)$, the function values follow a multivariate normal distribution prior $p(f) \sim N(f|0, K_{XX})$, where $f = \left(f(x_1), \dots, f(x_N)\right)^T \in R^N$, and where $K_{XX} \in R^{N \times N}$ with $[K_{XX}]_{ij} = k(x_i, x_j)$. The key property of Gaussian processes is that they encode functions that predict similar specificity values $f(x), f(x')$ for protein variants x, x' that are similar, where the similarity is encoded by the kernel $k(x, x')$. The key part of GP modelling is then to infer a kernel that measures the mutation's effects to the specificities.

Moreover we assumed an additive noise model

$$y = f(x) + \varepsilon, \qquad \varepsilon \sim N(0, \sigma_y^2),$$

where the observation was assumed to follow the true latent function $f(x)$ corrupted by zero-mean Gaussian noise with variance σ_y^2. We assumed a Gaussian process prior for the latent function f. A

dataset of N noisy specificity values into a vector $y \in R^N$ corresponding to the N protein mutant $X = (x_i)_{i=1}^N$ was collected, which then followed a Gaussian likelihood $p(y|f, \sigma_y^2 I)$.

We were interested in modelling the specificity of a new protein variant x_*. A Gaussian process defines a joint distribution over the observed values y of variants X, and the unknown function value $f(x_*)$ of the unseen variant x_*,

$$\begin{bmatrix} y \\ f(x_*) \end{bmatrix} = \begin{bmatrix} f + \epsilon \\ f(x_*) \end{bmatrix} \sim N\left(0, \begin{bmatrix} K_{XX} + \sigma_y^2 & k_{X*} \\ k_{*X} & k(x_*, x_*) \end{bmatrix}\right)$$

where $k_{X*} = k_{*X}^T \in R^N$ is a kernel vector with elements $k(x_i, x_*)$ for all $i = 1, \ldots, N$, and $k(x_*, x_*)$ is the self-kernel. A conditional distribution gives the posterior distribution of the specificity prediction as

$$f(x_*)|(X, y) \sim N(\mu(x_*), \sigma(x_*)^2),$$

where the prediction mean and variance are

$$\mu(x_*) = k_{*X}\left(K_{XX} + \sigma_y^2 I\right)^{-1} y,$$
$$\sigma(x_*)^2 = k(x_*, x_*) - k_{*X}\left(K_{XX} + \sigma_y^2 I\right)^{-1} k_{X*}.$$

Hence, in GP regression the specificity predictions $\mu(x_*) \pm \sigma(x_*)$ will come with uncertainty estimates in the form of Gaussian standard deviations. See Figure 1 for an illustration.

Graph kernel

Next, we considered how to compute the similarity function $k(x, x')$ between DERA variants. The 3D structural information of the protein variants were encoded as a contact map based on the PDB structure 1JCL, and their similarity was measured by the formalism of graph kernels (Vishwanathan et al. 2008).

Two residues were considered to be in contact if their closest atoms were within 5Å of each other in the PDB structure, which is illustrated in Figure 1. All variants of the same protein have the same length, with only different residues at mutating positions. We also assumed all variants to share the wild-type protein contact map.

In order to compare protein variants, we constructed a weighted decomposition kernel (WDK) (Menchetti et al. 2005) between two protein variants $x = (x_1 x_2 \cdots x_L)$ and $x' = (x_1' x_2' \cdots x_L')$ of length L,

$$k(x, x') = \sum_{i}^{L} S_{residue}(x_i, x_i') * S_{context}\big(N(x_i), N(x_i')\big)$$

$$= \sum_{i}^{L} S_{residue}(x_i, x_i') * \sum_{j}^{|N(x_i)|} S_{contact}\big(x_{ij}, x_{ij}'\big) * S_{residue}\big(x_j, x_j'\big)$$

where $N(x_i)$ defines the neighborhood of residue x_i, the $Sresidue(\cdot,\cdot)$ is a similarity kernel between selector residues, and $Scontext(\cdot,\cdot)$ is a similarity kernel between two residue contexts, and $Scontact(\cdot,\cdot)$ is a similarity kernel between contact pairs $x_{ij} := (x_i, x_j)$.

The kernel iterated over all positions i and compared for each of them their residues through a substitution matrix $Sresidue \in R^{20\times20}$. Furthermore, the similarity of the residues at each position was multiplied by the average similarity of the neighborhood, defined as a product of neighborhood residue substitutions and neighborhood contact substitution matrices $Scontact \in R^{400\times400}$. Hence, the kernel defined the similarity of two protein variants as the average position and neighbourhood similarity over all positions.

The above WDK kernel allowed us to compare the effects of multiple simultaneous mutations. However, as the wild-type *Ec* DERA protein structure was used for all protein variants, changes that the mutations may cause to the protein structure were not taken into consideration. This may have caused problems if mutations that alter the protein structure significantly were introduced – especially if many of them were introduced simultaneously. On the other hand, substitution matrices that have their basis in sequence comparisons, and take these effects into account to some extent as these kinds of mutations are usually highly destabilising and do not occur often in nature. In the next section, we will discuss how we utilise different substitution matrices with multiple kernel learning.

Substitution matrices and multiple kernel learning (MKL)

The BLOSUM substitution models have been a common choice for protein models (Giguère et al. 2013), while mixtures of substitution models were proposed by (Cichonska et al. 2017). BLOSUM matrices score amino acid substitutions by their appearances throughout evolution, as they compare

the frequencies of different mutations in similar blocks of sequences (Henikoff and Henikoff 1992). However, there are also different ways to score amino acids substitutions, such as chemical similarity and neighborhood selectivity (Tomii and Kanehisa M 1996). When the stability effects of mutations are evaluated, the frequency of an amino acid substitution in nature may not be the most important factor.

To take into account different measures of similarity between amino acids, we employed all 92 amino acid substitution matrices with no missing entries gathered from AAindex2 database (genome.jp/aaindex) (Kawashima et al. 2008) (See Table S3). We also gathered all 43 valid contact potential matrices from AAindex3 database (See Table S4). We ensured the positive definiteness of the substitution matrices by making the symmetric by averaging, truncating any possible negative eigenvalues, and scaling them to range $[0, 1]$. These substitution matrices were used for computing the residue and contact matrices. Finally, multiple kernel learning (MKL) was used to find an optimal combination of the base kernels of form

$$S_{residue} = \sum_{m} w^{m}_{residue} S^{m}_{residue}$$

$$S_{contact} = \sum_{m} w^{m}_{contact} S^{m}_{contact}$$

where $wresuidue \geq 0$ and $wcontact \geq 0$ are the substitution-specific weights. A zero weight indicates that the corresponding substitution matrix was not used. We observed empirically that the optimal kernel weights w_m tended to be sparse (See Figure S6).

The selected substitution matrices are listed in Figure S6. These matrices have different basis and through multiple kernel learning (MKL) our model learns which of these are important for inferring the specificity effects that mutations cause on different proteins. The Figure S6 illustrates this by showing the average weights of the base kernel matrices obtained via the multiple kernel learning.

Parameter Inference

The complete model has three parameter sets $\theta = (\sigma y, wres, wcon)$ for a total of 136 parameter values to infer, where the noise variance $\sigma_y^2 \in R_+$ represents the observation uncertainty, and the residue substitution and contact potential weights $w_{res} \in R^{92}$ and $w_{con} \in R^{43}$ parameterise the

optimal kernel model. In a Gaussian process regression model these can be tractably optimised by the marginal log likelihood

$$\log p(y|\sigma_y, w) = \log \int p(y|f, \sigma_y) p(f|w) df$$

$$\propto -\frac{1}{2} y^T \left(K_w + \sigma_y^2 I\right)^{-1} y - \frac{1}{2} \log |K_w + \sigma_y^2 I|$$

which automatically balances model fit (the square term) and the model complexity (the determinant) to avoid overfitting (Rasmussen and Willams 2006). The parameters can be optimised by maximising the above marginal log likelihood using gradient ascent, since the marginal likelihood can be differentiated analytically. We utilised a limited-memory projected quasi-Newton algorithm (minConf-TMP[2]), described by (Schmidt et al. 2009).

Validation

The accuracy of our predictions was evaluated using the same metrics that have been used by many others – correlation between the predicted and experimentally measured specificity values and the root mean square error. Marginal likelihood maximisation was used to infer model parameters and cross-validation was performed to evaluate the model performance on test data. In order to estimate the model generalisation performance, five-fold cross-validation was used. For the final predictions all training data was used.

We used the learnt Gaussian process model to screen all single, double and triple point mutations of *Ec* DERA *in silico*. All possible point mutations (total of 4,721) were screened, while we considered all combinations of the 67 unique observed mutation changes, resulting in 2,090 pairs and 42,026 triplets. In total we screened 48,837 *Ec* DERA variants *in silico*. From the top 50 best predicted variants, 18 mutants were manually chosen for mutagenesis and characterization experiments.

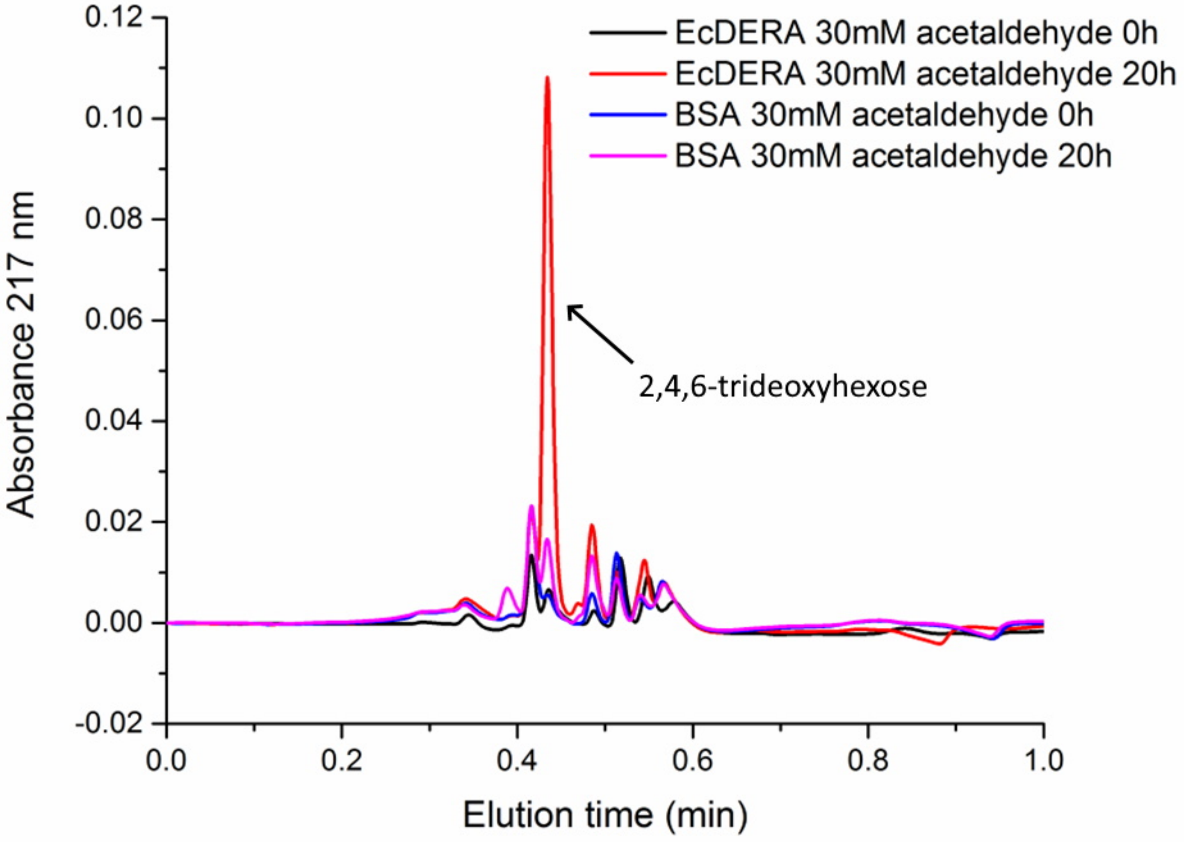

Fig S1 Setting up an LC assay for measuring the sequential acetaldehyde addition reaction by DERA. UPLC chromatogram was monitored at 217 nm for the product (2,4,6-trideoxyhexose) formation. Samples at time points 0 and 20 h containing either Ec DERA wild-type or BSA (as a negative control) are shown. This method was used to measure the acetaldehyde addition reaction of all the variants in this work.

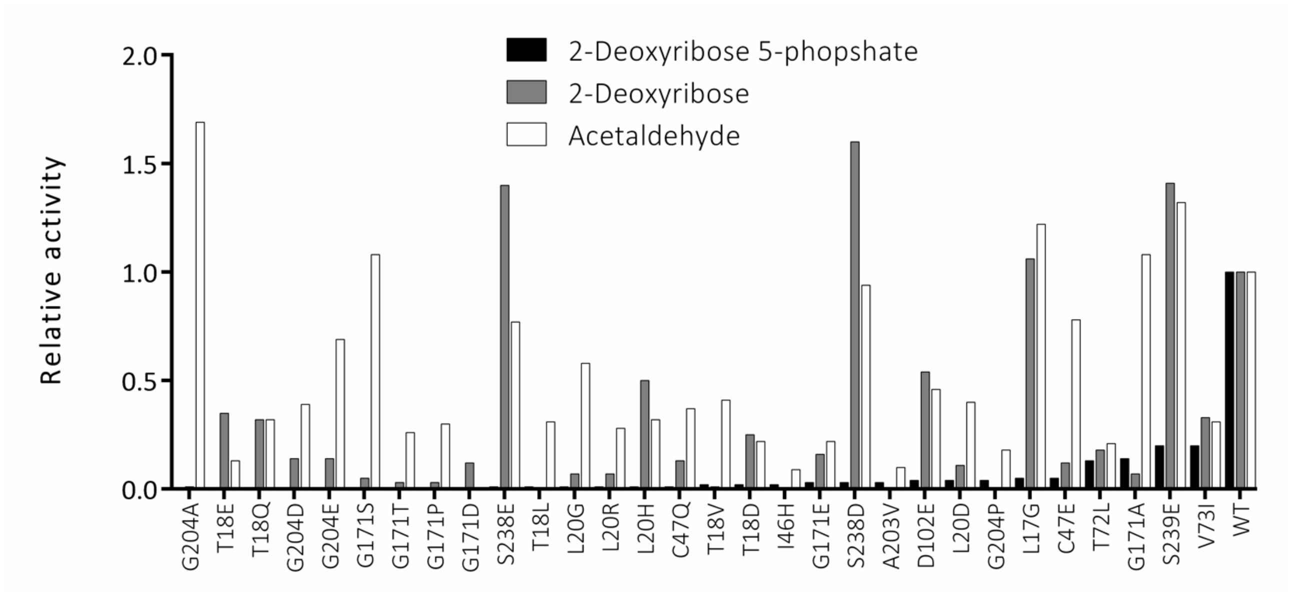

Fig S2 *Ec* DERA single point mutations variants having clearly lowered activity on the natural substrate DRP (20% or less DRP activity left). The 2-deoxyribose 5-phosphate (DRP) and 2-deoxyribose (DR) cleaving activities and acetaldehyde addition activities of *Ec* DERA variants are shown as relative activities compared to the wild-type *Ec* DERA (WT).

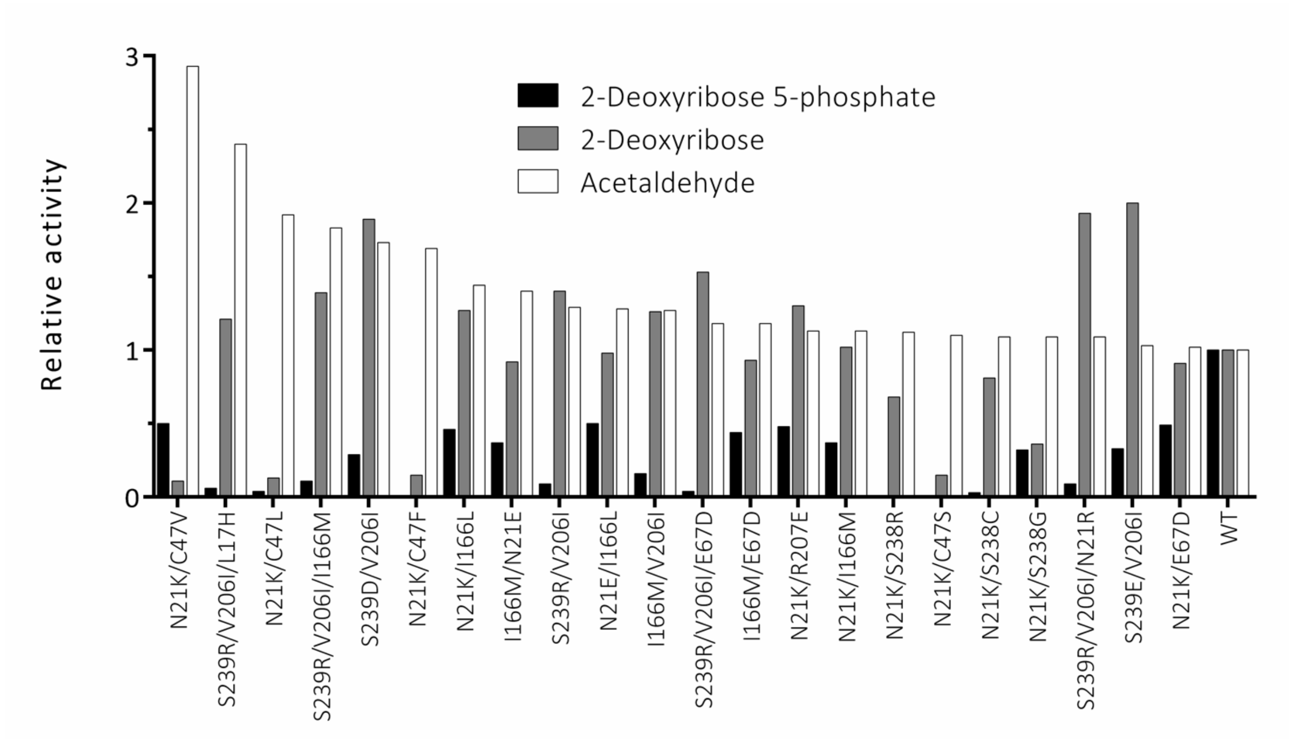

Fig S3 2-deoxyribose 5-phosphate (DRP) and 2-deoxyribose (DR) cleaving activities and acetaldehyde addition activities of *Ec* DERA variants containing two or three mutations, combined based on activity data or made through saturation mutagenesis. The specific activities are shown as relative activities compared to the wild-type DERA (WT). The most interesting results from the mutants containing two or three mutations are shown.

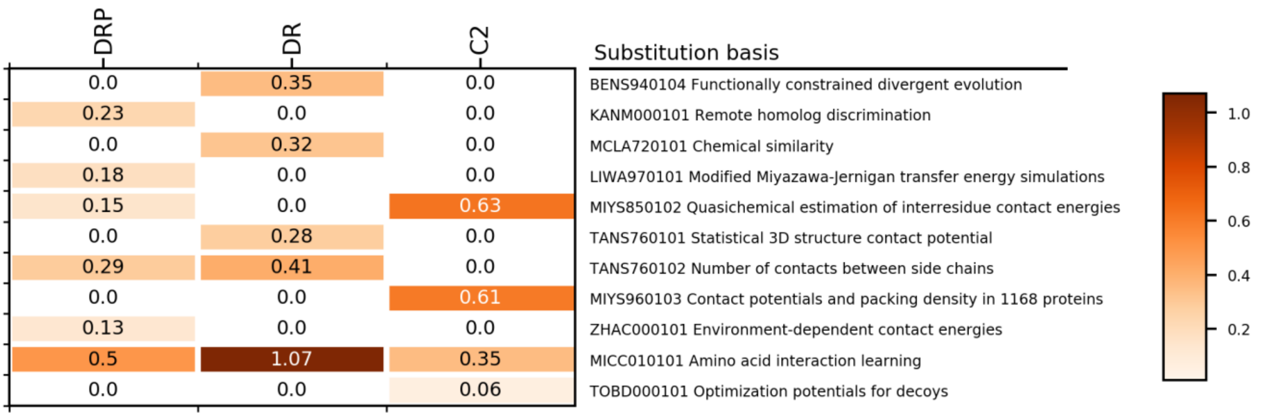

Fig S4 The trained substitution matrix weights against the three substrate specificities of DERA variants (columns DRP, DR, C2), a higher value having more predictive information towards the specificity. Only 11 substitution models (rows) had non-negative weights. For DR specificity prediction, amino acid interactions was the most important feature, while contact energies were the most informative feature for acetaldehyde specificity prediction.

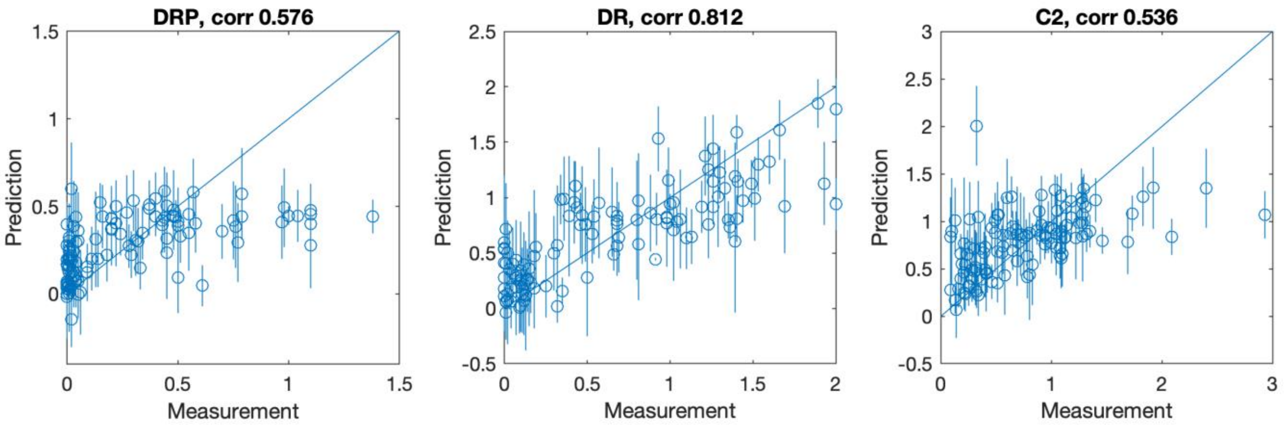

Fig S5 The 5-fold cross-validation test performance of the ML model on the characterization data from the first two mutagenesis rounds (altogether 131 *Ec* DERA mutants). The method achieves high correlation for DR specificity, and moderate correlation for DRP and acetaldehyde.

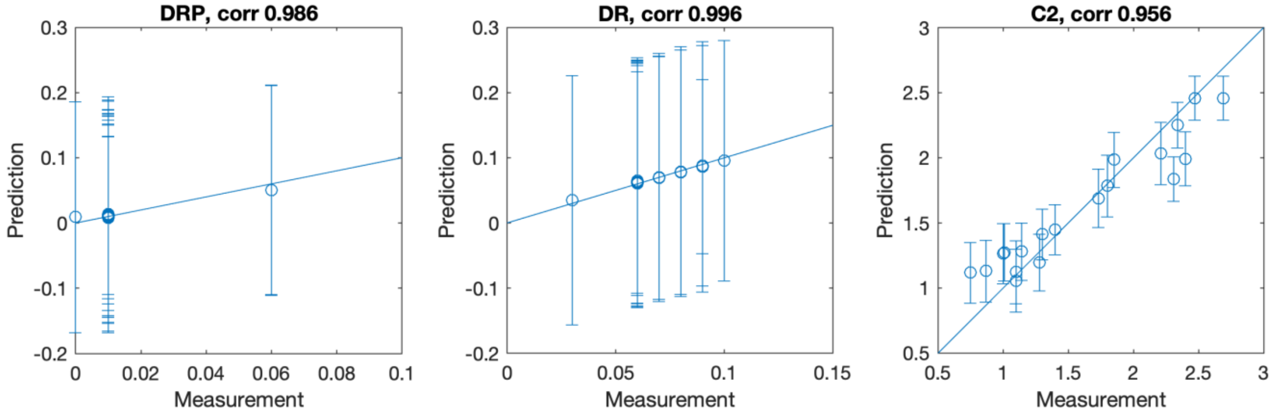

Fig S6 The empirical accuracy of the ML-guided 18 Ec DERA variants from the 3rd mutagenesis round, which were predicted to have low activity on DRP and DR, and maximal activity on acetaldehyde. Due to the removal of the DRP and DR specificity, the predicted performance is high. The 18 *Ec* DERA variants estimated to have a high acetaldehyde specificity had a correlation of 0.956 with the observed specificities.

Table S2 X-ray data collection and refinement statistics for the *Ec* DERA variants.

**Sample**	***Escherichia coli*** **DERA N21K**	***Escherichia coli*** **DERA N21K**	***Escherichia coli*** **DERA C47V/G204A/S239D**
**Ligand**	None	D -2-deoxyribose-5-phosphate	D -2-deoxyribose-5-phosphate
**Data collection**	ESRF ID30A-1	Rotating anode	DLS I24
**Wavelength (Å)**	0.9660	1.5418	0.9688
**Oscillation**	0.05°	1.0°	0.15°
**Exposure time (s)**	0.035	1200	0.010
**Number of images**	3300	180	1067
**Space group**	$P2_1$	$P2_1$	$P2_1$
a, b, c **(Å)**	60.5, 52.8, 80.7	61.9, 53.3, 81.2	62.2, 53.4, 80.8
**α, β, γ (°)**	90, 111.2, 90	90, 110.2, 90	90, 110.7, 90
**Resolution (Å)**	1.50–31.31 (1.50–1.55)	1.86–19.27 (1.86–1.97)	1.72–39.41 (1.72–1.78)
**Observations**	136475 (12275)	142080 (18431)	89923 (9206)
**Unique observations**	74834 (7258)	39746 (5999)	51138 (5131)
**Completeness**	98.3 % (96.0 %)	94.9 % (90.2 %)	96.9 % (97.8 %)
I / σ_i	10.0 (1.1)	10.5 (2.3)	8.5 (2.1)
$CC_{1/2}$	99.9 % (63.7 %)	99.4 % (71.0 %)	98.3 % (50.3 %)
R_{meas}	5.0 % (76.4 %)	12.5 % (66.4 %)	12.3 % (65.3 %)
R_{work}	15.9 %	16.1 %	18.2 %
R_{free}	18.7 %	20.9 %	22.7 %
**PDB code**	6Z9J	6Z9I	6Z9H

Numbers in the brackets are for the highest resolution shells.

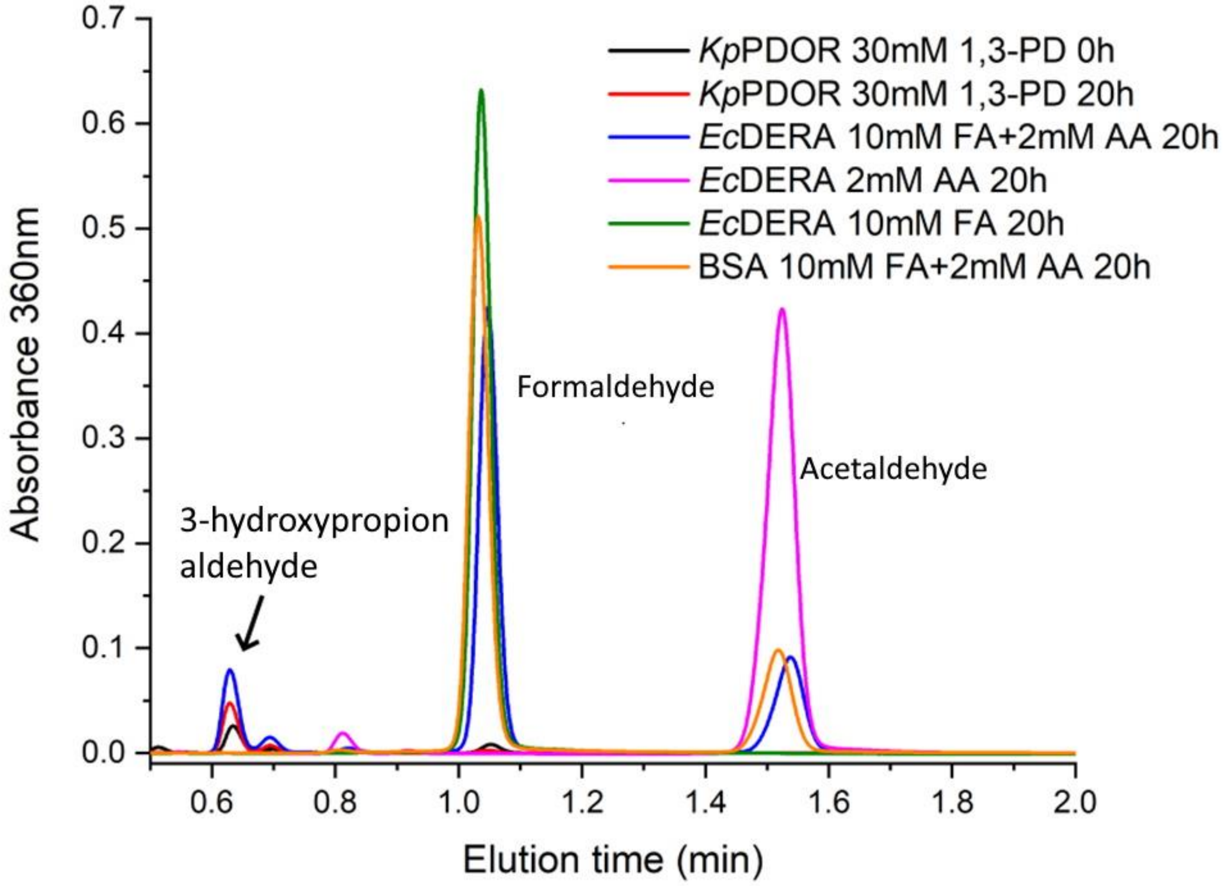

Fig S7 UPLC chromatogram at 360 nm for detection of the 2,4-DNPH derivatised products of DERA catalyzed addition of formaldehyde and acetaldehyde (blue line) and *Kp* PDOR catalysed oxidation of 1,3-propanediol (red line). The reaction products (3-hydroxypropionaldehyde) of both DERA activity and PDOR activity can be seen at around 0.6 min. Both DERA substrates, formaldehyde and acetaldehyde, are present after 20 h incubation, and they can be seen at around 1 min and 1.5 min elution time, respectively.

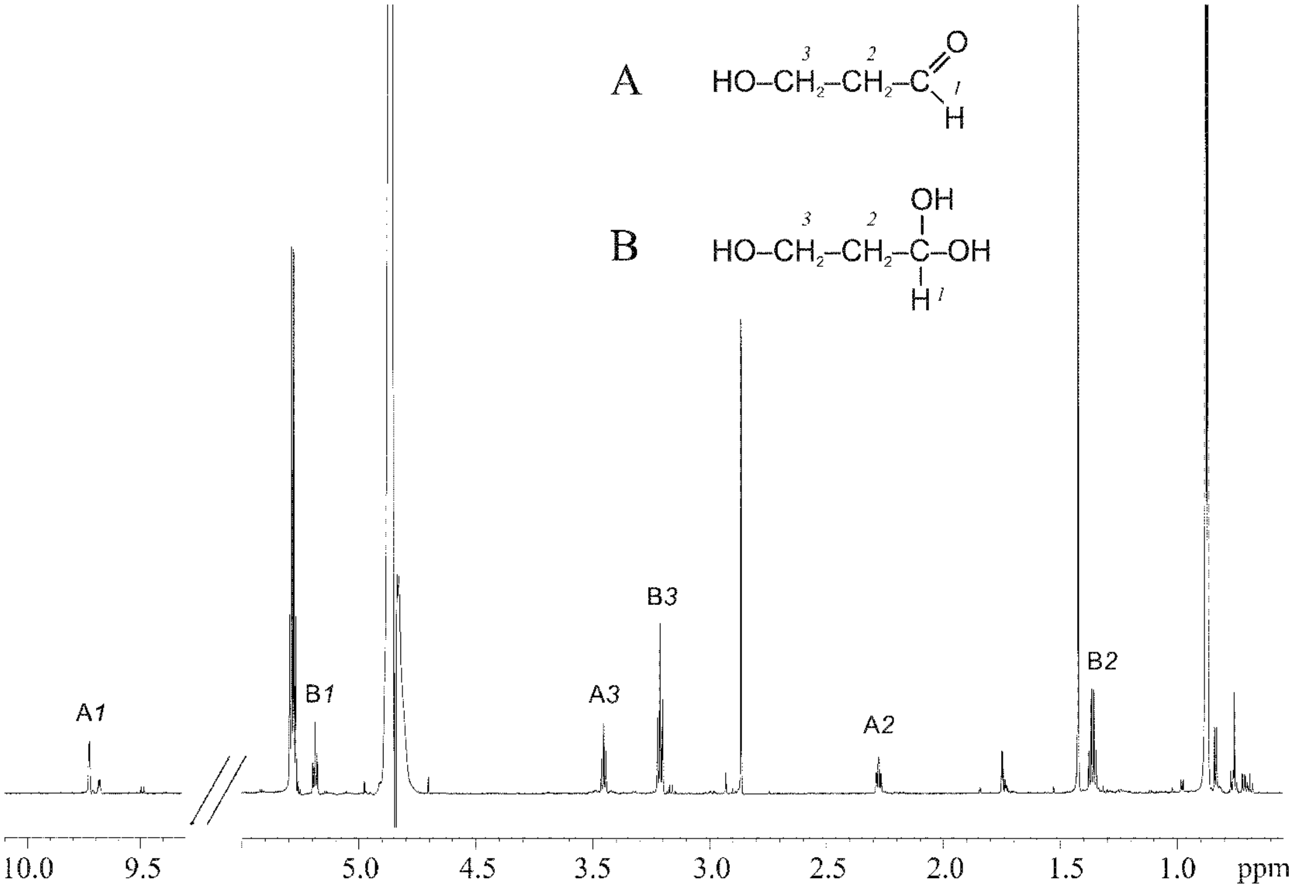

Fig S8 NMR identification of aldol addition product of formaldehyde and acetaldehyde by *Ec* DERA. 600 MHz ^{1}H NMR spectrum of the products 3-hydroxypropanal (A) and its hydrated form (B) of the DERA reaction of formaldehyde with acetaldehyde in 50 mM Na-phosphate buffer, pH 6.8, at 22°C. The assignments of the product signals to the ^{1}H atoms are indicated by the corresponding numbers.

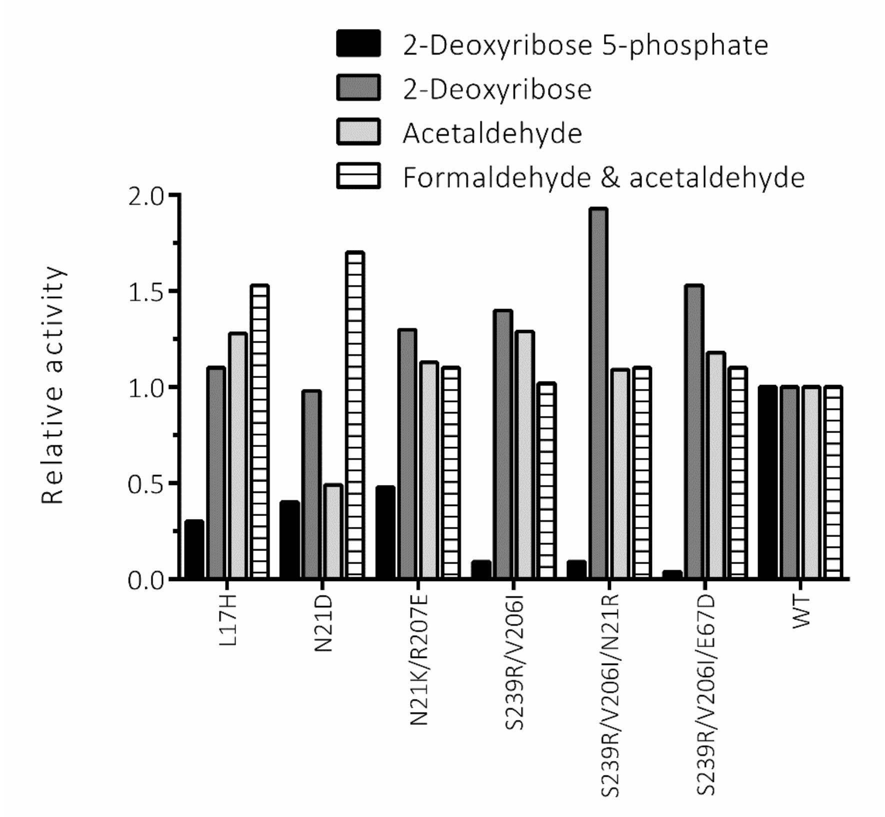

Fig S9 The best *Ec* DERA variants on formaldehyde and acetaldehyde aldol addition reaction. Activities on four different assays, i.e. on 2-deoxyribose 5-phosphate (DRP) and 2-deoxyribose (DR) cleavage, acetaldehyde addition, and on formaldehyde + acetaldehyde addition activity are shown as relative activities compared to the wild-type *Ec* DERA (WT).

Table S3 The residue substitution matrices used in the model from AAindex database.

List of 92 residue matrices from AAindex ver 9.1. (www.genome.jp/aaindex)
ALTS910101 The PAM-120 matrix (Altschul, 1991)
AZAE970101 The single residue substitution matrix from interchanges of
AZAE970102 The substitution matrix derived from spatially conserved motifs
BENS940101 Log-odds scoring matrix collected in 6.4-8.7 PAM (Benner et al., 1994)
BENS940102 Log-odds scoring matrix collected in 22-29 PAM (Benner et al., 1994)
BENS940103 Log-odds scoring matrix collected in 74-100 PAM (Benner et al., 1994)
BENS940104 Genetic code matrix (Benner et al., 1994)
BLAJ010101 Matrix built from structural superposition data for identifying potential
CROG050101 Substitution matrix computed from the Dirichlet Mixture Model
CSEM940101 Residue replace ability matrix (Cserzo et al., 1994)
DAYM780301 Log odds matrix for 250 PAMs (Dayhoff et al., 1978)
DAYM780302 Log odds matrix for 40 PAMs (Dayhoff et al., 1978)
DOSZ010101 Amino acid similarity matrix based on the sausage force
DOSZ010102 Normalised version of SM_SAUSAGE (Dosztanyi-Torda, 2001)
DOSZ010103 An amino acid similarity matrix based on the THREADER force field
DOSZ010104 Normalised version of SM_THREADER (Dosztanyi-Torda, 2001)
FEND850101 Structure-Genetic matrix (Feng et al., 1985)
FITW660101 Mutation values for the interconversion of amino acid pairs (Fitch, 1966)
GEOD900101 Hydrophobicity scoring matrix (George et al., 1990)
GIAG010101 Residue substitutions matrix from thermo/mesophilic to psychrophilic
GONG920101 The mutation matrix for initially aligning (Gonnet et al., 1992)
GRAR740104 Chemical distance (Grantham, 1974)
HENS920101 BLOSUM45 substitution matrix (Henikoff-Henikoff, 1992)
HENS920102 BLOSUM62 substitution matrix (Henikoff-Henikoff, 1992)
HENS920103 BLOSUM80 substitution matrix (Henikoff-Henikoff, 1992)
HENS920104 BLOSUM50 substitution matrix (Henikoff-Henikoff, 1992)
JOHM930101 Structure-based amino acid scoring table (Johnson-Overington, 1993)
JOND920103 The 250 PAM PET91 matrix (Jones et al., 1992)
JOND940101 The 250 PAM transmembrane protein exchange matrix (Jones et al., 1994)
KANM000101 Substitution matrix (OPTIMA) derived by maximizing discrimination between
KAPO950101 (Kapp et al., 1995)
KOLA920101 Conformational similarity weight matrix (Kolaskar-Kulkarni-Kale, 1992)
KOSJ950101 Context-dependent optimal substitution matrices for exposed helix
KOSJ950102 Context-dependent optimal substitution matrices for exposed beta
KOSJ950103 Context-dependent optimal substitution matrices for exposed turn
KOSJ950104 Context-dependent optimal substitution matrices for exposed coil
KOSJ950105 Context-dependent optimal substitution matrices for buried helix
KOSJ950106 Context-dependent optimal substitution matrices for buried beta
KOSJ950107 Context-dependent optimal substitution matrices for buried turn
KOSJ950108 Context-dependent optimal substitution matrices for buried coil
KOSJ950109 Context-dependent optimal substitution matrices for alpha helix
KOSJ950110 Context-dependent optimal substitution matrices for beta sheet
KOSJ950111 Context-dependent optimal substitution matrices for turn
KOSJ950112 Context-dependent optimal substitution matrices for coil
KOSJ950113 Context-dependent optimal substitution matrices for exposed residues
KOSJ950114 Context-dependent optimal substitution matrices for buried residues
KOSJ950115 Context-dependent optimal substitution matrices for all residues

LEVJ860101 The secondary structure similarity matrix (Levin et al., 1986)
LINK010101 Substitution matrices from an neural network model (Lin et al., 2001)
LUTR910101 Structure-based comparison table for outside other class (Luthy et al., 1991)
LUTR910102 Structure-based comparison table for inside other class (Luthy et al., 1991)
LUTR910103 Structure-based comparison table for outside alpha class (Luthy et al., 1991)
LUTR910104 Structure-based comparison table for inside alpha class (Luthy et al., 1991)
LUTR910105 Structure-based comparison table for outside beta class (Luthy et al., 1991)
LUTR910106 Structure-based comparison table for inside beta class (Luthy et al., 1991)
LUTR910107 Structure-based comparison table for other class (Luthy et al., 1991)
LUTR910108 Structure-based comparison table for alpha helix class (Luthy et al., 1991)
LUTR910109 Structure-based comparison table for beta strand class (Luthy et al., 1991)
MCLA710101 The similarity of pairs of amino acids (McLachlan, 1971)
MCLA720101 Chemical similarity scores (McLachlan, 1972)
MEHP950102 (Mehta et al., 1995)
MIYS930101 Base-substitution-protein-stability matrix (Miyazawa-Jernigan, 1993)
MIYT790101 Amino acid pair distance (Miyata et al., 1979)
MOHR870101 EMPAR matrix (Mohana Rao, 1987)
MUET010101 Non-symmetric substitution matrix (SLIM) for detection of homologous
MUET020101 Substitution matrix (VTML160) obtained by maximum likelihood estimation
MUET020102 Substitution matrix (VTML250) obtained by maximum likelihood estimation
NAOD960101 Substitution matrix derived from the single residue interchanges at spatially
NGPC000101 Substitution matrix (PHAT) built from hydrophobic and transmembrane regions
NIEK910101 Structure-derived correlation matrix 1 (Niefind-Schomburg, 1991)
NIEK910102 Structure-derived correlation matrix 2 (Niefind-Schomburg, 1991)
OGAK980101 Substitution matrix derived from structural alignments by maximizing entropy
OVEJ920101 STR matrix from structure-based alignments (Overington et al., 1992)
OVEJ920102 Environment-specific amino acid substitution matrix for alpha residues
OVEJ920103 Environment-specific amino acid substitution matrix for beta residues
OVEJ920104 Environment-specific amino acid substitution matrix for accessible
OVEJ920105 Environment-specific amino acid substitution matrix for inaccessible residues
PRLA000101 Structure derived matrix (SDM) for alignment of distantly related sequences
PRLA000102 Homologous structure dereived matrix (HSDM) for alignment of distantly
QUIB020101 STROMA score matrix for the alignment of known distant homologs
QU_C930101 Cross-correlation coefficients of preference factors
QU_C930102 Cross-correlation coefficients of preference factors
QU_C930103 The mutant distance based on spatial preference factor (Qu et al., 1993)
RIER950101 Hydrophobicity scoring matrix (Riek et al., 1995)
RISJ880101 Scoring matrix (Risler et al., 1988)
RUSR970101 Substitution matrix based on structural alignments of analogous proteins
RUSR970102 Substitution matrix based on structural alignments of remote homolous proteins
RUSR970103 Substitution matrix based on structural alignments of analogous and remote homolous
TUDE900101 isomorphicity of replacements (Tudos et al., 1990)
VOGG950101 (Vogt et al., 1995)
WEIL970101 WAC matrix constructed from amino acid comparative profiles (Wei et al., 1997)
WEIL970102 Difference matrix obtained by subtracting the BLOSUM62 from the WAC

Table S4 The contact potential matrices used in the model from AAindex database.

List of 43 contact potential matrices from AAindex ver 9.1. (www.genome.jp/aaindex)
BASU010101 Optimization-based potential derived by the modified perceptron criterion
BETM990101 Modified version of the Miyazawa-Jernigan transfer energy
BONM030101 Quasichemical statistical potential for the antiparallel orientation of
BONM030102 Quasichemical statistical potential for the intermediate orientation of
BONM030103 Quasichemical statistical potential for the parallel orientation of interacting
BONM030104 Distances between centers of interacting side chains in the antiparallel
BONM030105 Distances between centers of interacting side chains in the intermediate
BONM030106 Distances between centers of interacting side chains in the parallel
BRYS930101 Distance-dependent statistical potential (only energies of contacts within
KESO980101 Quasichemical transfer energy derived from interfacial regions of
KESO980102 Quasichemical energy in an average protein environment derived from interfacial
KOLA930101 Statistical potential derived by the quasichemical approximation
LIWA970101 Modified version of the Miyazawa-Jernigan transfer energy
MICC010101 Optimization-derived potential
MIRL960101 Statistical potential derived by the maximization of the harmonic mean of Z
MIYS850102 Quasichemical energy of transfer of amino acids from water to the protein
MIYS850103 Quasichemical energy of interactions in an average buried environment
MIYS960101 Quasichemical energy of transfer of amino acids from water to the protein
MIYS960102 Quasichemical energy of interactions in an average buried environment
MIYS960103 Number of contacts between side chains derived from 1168 x-ray protein
MIYS990106 Quasichemical energy of transfer of amino acids from water to the protein
MIYS990107 Quasichemical energy of interactions in an average buried environment
MOOG990101 Quasichemical potential derived from interfacial regions of protein-protein
SIMK990101 Distance-dependent statistical potential (contacts within 0-5 Angstrooms)
SIMK990102 Distance-dependent statistical potential (contacts within 5-7.5 Angstrooms)
SIMK990103 Distance-dependent statistical potential (contacts within 7.5-10 Angstrooms)
SIMK990104 Distance-dependent statistical potential (contacts within 10-12 Angstrooms)
SIMK990105 Distance-dependent statistical potential (contacts longer than 12 Angstrooms)
SKOJ000101 Statistical quasichemical potential with the partially composition-corrected
SKOJ000102 Statistical quasichemical potential with the composition-corrected pair scale
SKOJ970101 Statistical potential derived by the quasichemical approximation
TANS760101 Statistical contact potential derived from 25 x-ray protein structures
TANS760102 Number of contacts between side chains derived from 25 x-ray protein structures
THOP960101 Mixed quasichemical and optimization-based protein contact potential
TOBD000101 Optimization-derived potential obtained for small set of decoys
TOBD000102 Optimization-derived potential obtained for large set of decoys
VENM980101 Statistical potential derived by the maximization of the perceptron criterion
ZHAC000101 Environment-dependent residue contact energies (rows = helix, cols = helix)
ZHAC000102 Environment-dependent residue contact energies (rows = helix, cols = strand)
ZHAC000103 Environment-dependent residue contact energies (rows = helix, cols = coil)
ZHAC000104 Environment-dependent residue contact energies (rows = strand, cols = strand)
ZHAC000105 Environment-dependent residue contact energies (rows = strand, cols = coil)
ZHAC000106 Environment-dependent residue contact energies (rows = coil, cols = coil)

Supplementary references

Cichonska A, Ravikumar B, Parri E, Timonen S, Pahikkala T, Airola A, Wennerberg K, Rousu J, Aittokallio T (2017) Computational-experimental approach to drug-target interaction mapping: A case study on kinase inhibitors. PLoS Comput Biol 13:1–28 . doi: 10.1371/journal.pcbi.1005678

Giguère S, Marchand M, Laviolette F, Drouin A, Corbeil J (2013) Learning a peptide-protein binding affinity predictor with kernel ridge regression. BMC Bioinformatics. doi: 10.1186/1471-2105-14-82

Henikoff S, Henikoff JG (1992) Amino acid substitution matrices from protein blocks. Proc Natl Acad Sci U S A 89:10915–9

Kawashima S, Pokarowski P, Pokarowska M, Kolinski A, Katayama T, Kanehisa M (2008) AAindex: Amino acid index database, progress report 2008. Nucleic Acids Res 36:202–205 . doi: 10.1093/nar/gkm998

Menchetti S, Costa F, Frasconi P (2005) Weighted decomposition kernels. In: Proceedings of the 22nd international conference on Machine learning. ACM, pp 585–592

Rasmussen CE, Willams CKI (2006) Gaussian processes for machine learning. MIT Press ISBN:026218253X

Schmidt M, Berg E Van Den, Friedlander MP, Murphy K (2009) Optimizing Costly Functions with Simple Constraints : A Limited-Memory Projected Quasi-Newton Algorithm. Icml 5:456–463

Tomii K, Kanehisa M (1996) Analysis of amino acid indices and mutation matrices for sequence comparison and structure prediction of proteins. Protein Eng 9:27–36

Vishwanathan SVN, Borgwardt KM, Kondor IR, Schraudolph NN (2008) Graph Kernels. 11:1201–1242
